# Lepidiline-Derived Imidazole-2(3*H*)-Thiones: (3+2)-Cycloadditions vs. Nucleophilic Additions in Reactions with Fluorinated Nitrile Imines

**DOI:** 10.3390/molecules30193851

**Published:** 2025-09-23

**Authors:** Wiktor K. Poper, Kamil Świątek, Katarzyna Urbaniak, Barbara Olszewska, Marcin Jasiński

**Affiliations:** 1 Department of Organic and Applied Chemistry, Faculty of Chemistry, University of Lodz, 91403 Łódź, Poland; 2 Doctoral School of Exact and Natural Sciences, The University of Lodz, 90237 Łódź, Poland

**Keywords:** spiro-heterocycles, nitrile imines, imidazoles, (3+2)-cycloadditions, lepidilines, fluorinated products, alkaloids

## Abstract

Two series of imidazole-2(3*H*)-thiones inspired by naturally occurring lepidiline alkaloids, bearing either one or two benzyl-type substituents located at the N(1)/N(3) atoms, respectively, were prepared and examined in reactions with in situ generated *C*-trifluoromethyl-*N*-aryl nitrile imines. *N*,*N*-Dibenzylated imidazole-2-thiones served exclusively as C=S dipolarophiles to afford hitherto unknown CF_3_-functionalized spiro [1,3,4-thiadiazole-5,2′-imidazole] derivatives formed through the (3+2)-cycloaddition pathway. In contrast, the enolizable *N*-monobenzylated imidazole-2-thiones provided acyclic products, i.e., hydrazonothioates, resulting from nucleophilic addition of the respective en(thio)late onto the *C*-termini of the 1,3-dipole. The presented results extend the scope of both fluorinated products available via trapping of the in situ generated CF_3_-nitrile imines as well as synthetic analogues of lepidilines. In addition, spectroscopic analysis of the obtained products and the known related systems revealed ^13^C NMR chemical shifts attributed to the *C*-(CF_3_) atom as useful probes to differentiate the open-chain hydrazonothioates (δ = 112–120), 2,2-diaryl/dialkyl-2,3-dihydro-1,3,4-thiadiazoles (δ = 130–145), and more strained spiro-1,3,4-thiadiazole derivatives (δ = 166–170) reported herein.

## 1. Introduction

**Figure 1:**
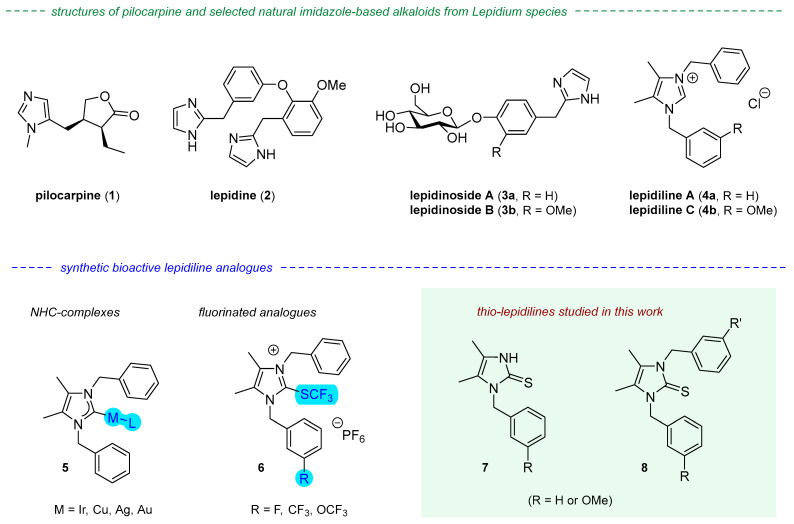
Structures of pilocarpine (**1**) and selected imidazole-based alkaloids **2**–**4** found in *Lepidium* species, synthetic bioactive lepidiline analogues **5** and **6**, and the enolizable and non-enolizable thio-lepidilines **7** and **8**, respectively, studied herein.

There is increasing interest in the chemistry of imidazole alkaloids recognized as compounds of various structures and numerous roles in biological processes, and in certain cases, they are applied for medicinal needs [1,2,3,4]. Although the majority of known imidazole alkaloids originate from marine organisms, terrestrial flora also offer a variety of imidazole-based materials. For example, a lactone-functionalized imidazole alkaloid pilocarpine (**1**), first isolated from north Brazilian plant Maranham Jaborandi (*Pilocarpus microphyllus*), is an effective agent for treatment of so-called ‘dry mouth’ (xerostomia) as well as medication to treat glaucoma in order to reduce intraocular pressure (pressure inside the eye) (Figure 1) [5]. *Lepidium* genome, widely distributed in South America, is considered another valuable source of imidazole-based alkaloids. Thus, non-ionic dimeric structures such as lepidine (**2**) as well as monomeric lepidinoside A (**3a**, R = H) and lepidinoside B (**3b**, R = OMe) functionalized with β-d-galactopyranose moiety were identified as secondary metabolites in annual herb called garden cress (*Lepidium sativum*) [6,7]. On the other hand, several ionic *N*,*N*-dibenzylated imidazolium alkaloids such as lepidiline A and lepidiline C (**4a**,**b**; R = H or OMe, respectively) were identified in Maca root (*Lepidium meyenii*) [8,9,10], a known nutrient food rich in vitamins, minerals, and proteins [11,12].

Notably, natural lepidilines exhibit promising anticancer properties [8], and for this reason, modification of their structures is considered an attractive strategy for the preparation of new bioactive compounds with desired bioactivity profiles. For example, in a series of independent work by Tacke, Kerr, and Tóth groups, lepidiline A demonstrated a suitable starting material for preparation of *N*-heterocyclic carbene (NHC) complexes with several metal ions, of which silver(I) and gold(I) complexes were demonstrated to be remarkably cytotoxic against ovarian and uterine, as well as breast cancer cells (Figure 1) [13,14,15]. Furthermore, our work demonstrated that introduction of the fluorine atom or fluorinated groups such as CF_3_ and OCF_3_ at *meta* position of the peripheral benzylic substituents in lepidiline [16], as well as functionalization of the central imidazole ring with the SCF_3_ moiety [17], remarkably amplifies anticancer activity of the parent alkaloid. Hence, further study towards fluorinated lepidilines of various substitution patterns is of general interest.

**Scheme 1:**
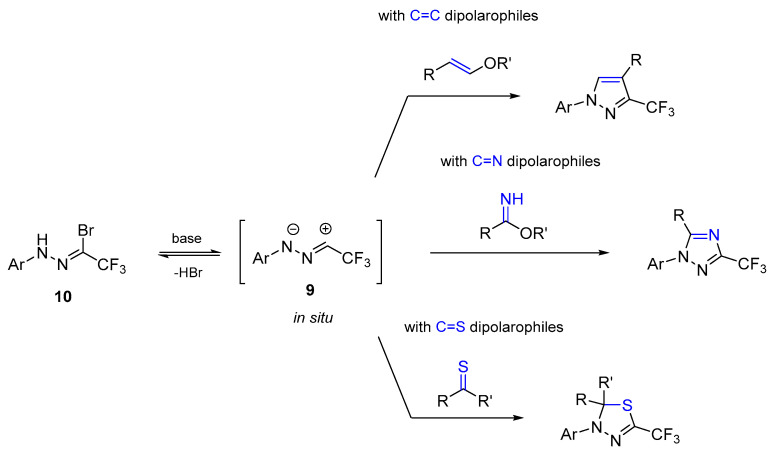
Generation of CF_3_-nitrile imines **9** through base-induced dehydrohalogenation of hydrazonoyl bromides **10**, and exemplary 1,3-dipolar cycloadditions of **9** with selected C=C, C=N, and C=S dipolarophiles leading to pyrazoles, 1,2,4-triazoles, and 1,3,4-thiadiazoles, respectively.

In a series of recent work, the CF_3_-functionalized nitrile imines of type **9**, formally derived from trifluoroacetonitrile, were demonstrated to be highly useful building blocks to access various fluorinated heterocyclic systems of general importance for pharmaceutical and agrochemical applications (Scheme 1) [18,19,20,21,22]. The mentioned 1,3-dipoles **9** can be easily generated in situ through base-mediated dehydrohalogenation of the respective hydrazonoyl precursors such as bromides **10** [23], and in the presence of suitable dipolarophile, they smoothly undergo Huisgen cycloaddition reactions to give five-membered products. For instance, highly chemo- and regioselective (3+2)-cycloadditions using the C=C (or C≡C), C=N, and the C=S dipolarophilic reaction partners were developed to afford valuable CF_3_-functionalized pyrazoles [24,25,26,27], 1,2,4-triazoles [28,29,30], and 1,3,4-thiadiazole derivatives [31,32,33], respectively.

Taking into account the unusually high reactivity of the C=S group in 1,3-dipolar cycloaddition reactions (for this reason, thiocarbonyls are often called as *superdipolarophiles* [34,35]), in continuation of our quest towards fluorinated lepidilines and related imidazole derivatives [36,37,38], the thioxo-functionalized analogues, i.e., enolizable and non-enolizable imidazole-2(3*H*)-thiones **7** and **8** (Figure 1), respectively, were prepared and examined in reactions with in situ-generated CF_3_-nitrile imines **9**. Here we report different reaction outcomes observed for reactions of the selected imidazole-2-thiones; whereas enolizable substrates of type **7** provided exclusively acyclic products (i.e., hydrazonothioates) formed by nucleophilic addition of the respective thiolates onto the *C*-termini of the 1,3-dipole, the non-enolizable 1,3-dibenzyl-imidazole-2-thiones **8** afforded solely the expected spiro [1,3,4-thiadiazole-5,2′-imidazole] derivatives resulting from the (3+2)-cycloaddition of the 1,3-dipole and the C=S group of dipolarophile **8**. The scope and limitations of both developed reactions was also checked.

## 2. Results and Discussion

**Scheme 2:**
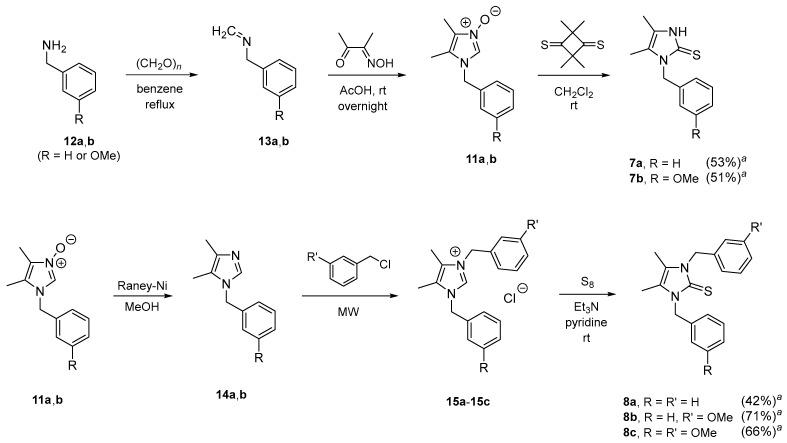
Synthesis of imidazole-2-thiones **7a**,**b** and **8a**–**8c** employing imidazole *N*-oxides **11** as key intermediates: *^a^* overall yield for 3 steps.

The required imidazole-2-thiones **7** and **8** are readily available building blocks that can be prepared using the corresponding nitrone-like 2-unsubstitutied imidazole *N*-oxides **11** recognized as convenient precursors for various imidazole-based systems [39,40,41]. Following the general protocol depicted in Scheme 2, two selected enolizable imidazole derivatives bearing benzyl (**7a**) or 3-methoxybenzyl (**7b**) substituent located at the N(1) of the imidazole ring were prepared through the one-pot telescopic approach starting from the respective benzylamines **12** (see, Supporting Information in Supplementary File S1), by treatment of **12** with solid paraformaldehyde under reflux, followed by acid-induced (in glacial AcOH [36]) condensation of the first formed formaldimines **13** with diacetyl monoxime to give *N*-oxides **11a** and **11b**. Sulfur-transfer with 2,2,4,4-tetramethylcyclobutane-1,3-dithione [42] provided the desired enolizable imidazole-2-thiones **7a** and **7b**, which were isolated in fair overall yield of 53% and 51% (for 3 steps), respectively. With two intermediate imidazole *N*-oxides **11a** and **11b** in hand, the non-enolizable series of imidazole-2-thiones **8a**–**8c** was synthesized in a one-pot manner starting with deoxygenation of **11** with Raney-nickel, in MeOH, at room temperature, followed by microwave-activated *N*-benzylation of the imidazole ring with selected benzyl chlorides (R′ = H or OMe) to give the corresponding imidazolium chlorides **15**. Subsequent sulphuration of the in situ-generated *N*-heterocyclic carbenes derived from salts **15** with elemental sulfur in pyridine afforded desired products of type **8**. Also, in this case the overall yield of isolated products **8a**, **8b**, and **8c** was satisfactory (42%, 71%, and 66%, respectively; for three steps from imidazole *N*-oxides **11**).

**Scheme 3:**
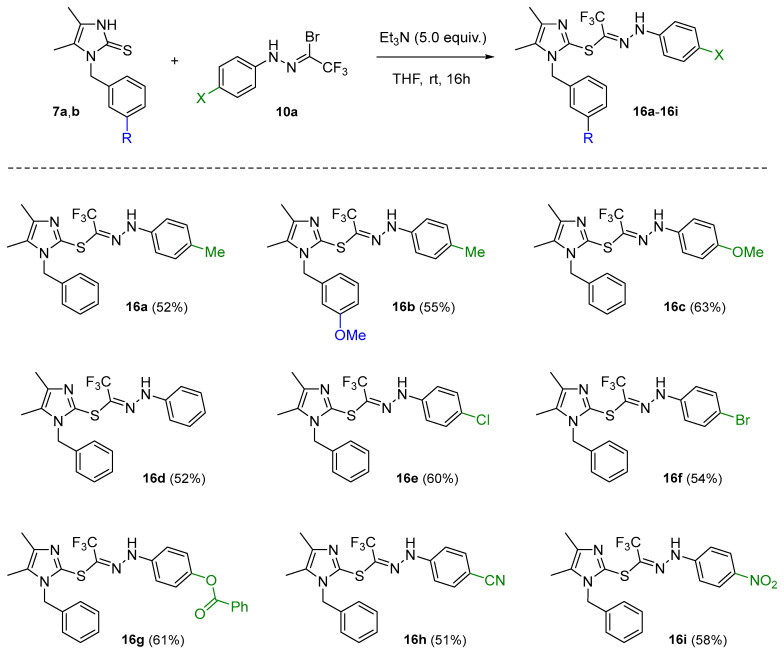
Synthesis of hydrazonothioates **16a**–**16i** derived from enolizable imidazole-2-thiones **7a** and **7b**: scope of hydrazonoyl bromides **10**.

A series of CF_3_-nitrile imine precursors, i.e., hydrazonoyl bromides **10**, was prepared following the general literature protocols by NBS-mediated bromination [31] of the azomethine group in trifluoroacetaldehyde arylhydrazones [43]. Based on our experience in base-induced in situ generation of nitrile imines **9** and their trapping with dipolarophiles [24,25,26,44,45] as well as other bifunctional reagents in solution [46,47], but also under solvent-free mechanochemical conditions [48,49], the initial experiments were carried out using slight excess imidazole-2-thione **7a** (1.2 equiv.), and *N*-tolyl-functionalized bromide **10a** (1.0 mmol) selected as a model precursor, in dry THF solution, in the presence of three-fold excess Et_3_N (Scheme 3). To our delight, slow consumption of the starting imidazole-2-thione was observed at room temperature, and after overnight stirring, the reaction was complete. Whereas increasing excess of triethylamine to 5 equiv. slightly accelerated the studied transformation, replacement of THF by toluene or by dichloromethane showed no positive effects on the reaction progress. Also, increase in the reaction temperature (THF, up to 65 °C) provided more complex reaction mixtures, presumably due to decomposition of the first formed product. Removal of the solid triethylammonium bromide by filtration followed by purification of the crude products on chromatography column provided solid material, which was recrystallized from petroleum ether/dichloromethane mixture to give colorless crystalline **16a** in 52% yield. Whereas the ESI-MS measurements supplemented by combustion analysis confirmed the expected molecular formula as C_21_H_21_F_3_N_4_S, analysis of spectroscopic data of the isolated material suggested the open-chain structure of **16a** (i.e., the respective hydrazonothioate resulting from nucleophilic addition) rather than the isomeric (3+2)-cycloaddition product formed by the attack onto the C=S bond. In particular, in the ^13^C NMR spectrum of **16a** the diagnostic quartet (^2^*J*_C-F_ = 37.6 Hz) attributed to the *C*-(CF_3_) atom was found at a remarkably higher field of δ = 113.2 in comparison to absorptions observed for typical 2,2-(hetero)diaryl- and 2,2-dialkyl-2,3-dihydro-1,3,4-thiadiazole derivatives, usually between δ = 130–145 [31,32,33].

An analogous result was observed for the reaction of *m*-methoxybenzyl-functionalized derivative **7b** with the model CF_3_-nitrile imine **9a**; also in that case, the corresponding hydrazonothioate **16b** was formed as a single product, which was isolated as an analytically pure oil in comparable yield of 55% (Scheme 3). Next, a series of hydrazonoyl bromides **10b**–**10h** was involved into the study and examined in reactions with imidazole-2-thione **7a** under standard reaction conditions. In all the experiments, the exclusive thiophilic addition of **7a** onto positively charged C atom of the nitrile imine **9** was observed to afford hydrazonothioates **10c**–**10i** in moderate yield (51–63%), irrespectively of the electronic nature of the substituent located at the *para* position of the *N*-phenyl group in hydrazonoyl bromide **10**.

**Scheme 4:**
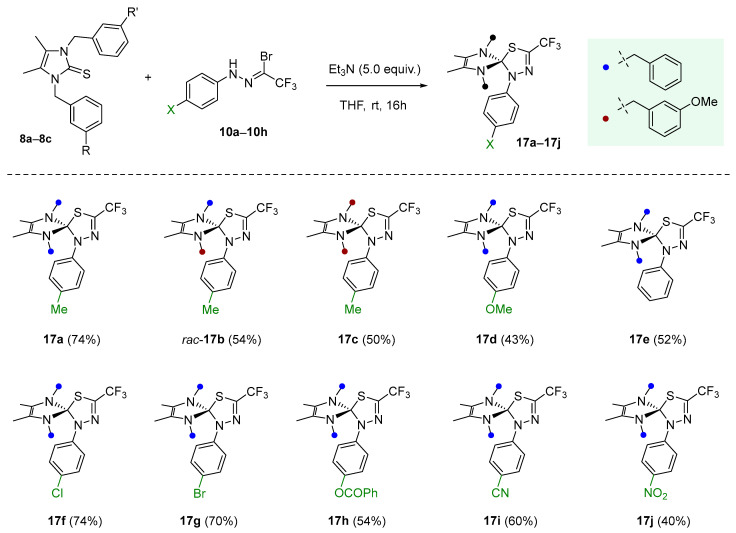
Synthesis of spiro [1,3,4-thiadiazole-5,2′-imidazole] derivatives **17a**–**16j** derived from non-enolizable imidazole-2-thiones **8a**–**8c**: scope of hydrazonoyl bromides **10**.

In continuation of the study, 1,3-dibenzyl-4,5-dimethylimidazole-2-thione (**8a**) was examined in reaction with model CF_3_-nitrile imine **9a** under the analogous reaction conditions. The consumption of starting materials along with the formation of a single product was observed within 6 h (by TLC monitoring), and after removal of the solvents followed by standard column chromatography, the expected (3+2)-cycloadduct **17a** (74%) was isolated as a colorless solid (Scheme 4). The structure of **17a** was confirmed on the basis of spectroscopic analysis; for example, in the ^1^H NMR spectrum, a set of characteristic signals attributed to the C_2_-symmetric imidazole unit was located at δ = 2.06 (6H, 2 Me) and δ = 5.08 (4H, 2 CH_2_Ph), while absorptions found at δ = 2.16 (3H, Me) and between δ = 6.55–7.11 (14H) revealed the presence of all three aromatic substituents. More importantly, in the ^13^C NMR spectrum the diagnostic absorption attributed to the C(2) atom of the 1,3,4-thadiazole ring was found at δ = 167.2 (^2^*J*_C-F_ = 33.3 Hz), indicating the formation of a strained system, while absorption at δ = −66.5 in the ^19^F NMR spectrum denoted the presence of a single CF_3_ group. Further tests including ESI-MS measurements and combustion analysis confirmed the molecular formula of **17a** as C_28_H_27_F_3_N_4_S and the analytical purity of the sample.

Imidazole-2-thiones bearing one (**8b**) or two *m*-methoxybenzyl (**8c**) groups were also reacted with CF_3_-nitrile imine **9a** to afford (3+2)-cycloadducts *rac*-**17b** and **17c**, albeit partial decomposition of these products was observed during purification on column, and the spectroscopically pure samples were isolated in lower yield (54% and 50%, respectively). Finally, a series of variously substituted nitrile imines were examined, and in all of the cases the expected spiro products were formed. Notably, the presence of such synthetically useful substituents and functional groups as methoxy, halogens (Cl and Br), ester (OCOPh), as well as strongly electron-withdrawing cyano and nitro moieties were tolerated, and the desired materials **17d**–**17j** were isolated in fair 50–74% yield (Scheme 4).

**Figure 2:**
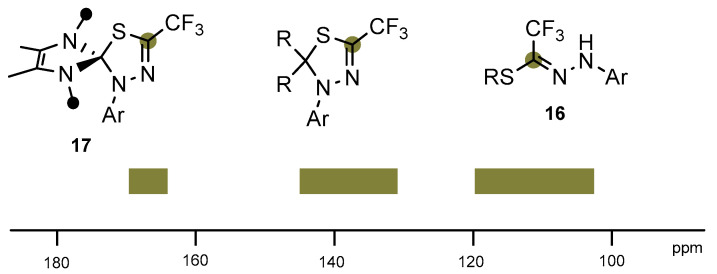
The diagnostic ^13^C NMR chemical shifts attributed to the *C*-(CF_3_) atom in known CF_3_-functionalized 2,2-dialkyl/diaryl-2,3-dihydro-1,3,4-thiadiazoles [31,32,33,50], lepidiline-derived hydrazonothioates **16** and spiro-1,3,4-thiadiazole derivatives **17** reported in this work.

Some time ago, a series of thiocarbonyl compounds was examined in reactions with CF_3_-nitrile imines of type **9**. Thus, (cyclo)aliphatic [31] and (hetero)aromatic thioketones [32], monomeric thiochalcones [33] as well as perfluorinated secondary thioamides [50] provided the expected 5-membered products, namely 2,2-disubstituted 2,3-dihydro-1,3,4-thiadiazoles, which were fully characterized by spectroscopic methods. In all of these cases, in the ^13^C NMR spectra the diagnostic absorption attributed to the C(5) atom (q, ^2^*J*_C-F_ ≈ 37–43 Hz) linked with the CF_3_ group was found between δ = 130–145 (Figure 2).

In the present work, the thiourea-type lepidiline analogues **7** and **8** provided different products depending on the substitution pattern; whereas enolizable substrates afforded hydrazonothioates **16**, the non-enolizable starting materials yielded solely the corresponding *spiro* products **17**. These structural differences can be observed in the ^13^C NMR spectra as the analogous quartet signal is either remarkably high-field (unhindered adducts) or down-field shifted (more strained spiro[imidazo-1,3,4-thiadiazoles]), respectively (Figure 2). Thus the ^13^C NMR absorption of the *C*(CF_3_) atom can be considered a useful probe to easily differentiate the type of products formed in reactions between thiocarbonyl compounds and the CF_3_-nitrile imines.

**Scheme 5:**
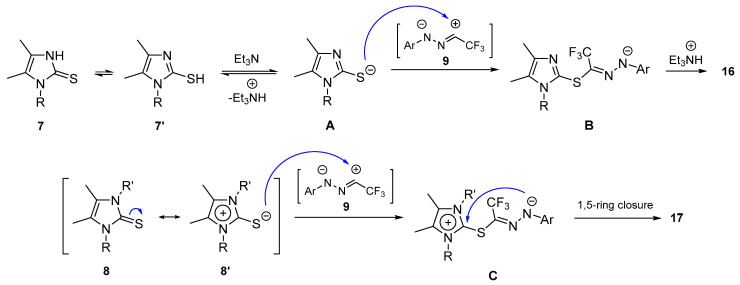
The proposed mechanisms for the formation of hydrazonothioates **16** and (3+2)-cycloadducts **17**.

The formation of hydrazonothioates **16** and spiro (3+2)-cycloadducts **17** also deserve a brief comment; in the former case, the starting enolizable imidazole-2-thione **7** presumably suffer deprotonation under the applied reaction conditions to give highly nucleophilic en(thio)late intermediate **A** (Scheme 5). The formed anion smoothly adds to the *C*-termini of the nitrile imine **9** to give **B**. Subsequent protonation of the negatively charged N atom in nicely stabilized **B** leads to sterically unhindered product **16**. In the case of non-enolizable imidazole-2-thiones **8**, no corresponding thiolate anion operates; taking into account known remarkable polarization of the carbon–sulfur bond in imidazole-2-thiones [51], we tentatively propose that the reaction initiates thiophilic addition of nitrile imine onto **8′**, leading to zwitterionic intermediate of type **C**. This first formed adduct undergoes 1,5-ring closure to afford final sterically demanding (3+2)-cycloadduct **17** in a fully regioselective manner.

## 3. Materials and Methods

### 3.1. Chemical Synthesis General Methods

Experimental procedures: Solvents and chemicals were purchased and used as received. Products were purified by standard column chromatography (CC) on silica gel (230–400 mesh). Melting points were determined in capillaries with a MEL-TEMP II apparatus (Laboratory Devices, Holliston, MA, USA) or with a Stuart SMP30 apparatus (Bibby Scientific Ltd., Stone, Staffordshire, UK) with automatic temperature monitoring, and are uncorrected. The NMR spectra were taken on a Bruker AVIII instrument (^1^H at 600 MHz, ^13^C at 151 MHz, and ^19^F at 565 MHz) (Bruker BioSpin AG, Fällanden, Switzerland). Chemical shifts are reported relative to solvent residual peaks; for CDCl_3_: ^1^H NMR: δ = 7.26, ^13^C NMR: δ = 77.16 or to CFCl_3_ (^19^F NMR: δ = 0.00) used as external standard. The IR spectra were measured with an Agilent Cary 630 FTIR spectrometer (Agilent Technologies, Santa Clara, CA, USA), in neat. MS (ESI) were taken with a Varian 500-MS LC Ion Trap (Varian, Inc., Walnut Creek, CA, USA), and high resolution MS (ESI-TOF) measurements were taken with a Waters Synapt G2-Si mass spectrometer (Waters Corporation, Milford, MA, USA). Combustion analyses were performed with a Vario EL III (Elementar Analysensysteme GmbH, Langenselbold, Germany) instrument.

Starting materials: Hydrazonoyl bromides **10** were prepared by reaction of the corresponding trifluoroacetaldehyde arylhydrazones with NBS, according to general protocol [31], while the required fluoral hydrazones were obtained by condensation of aqueous fluoral hydrate (~75% in H_2_O) with commercial arylhydrazines [43]. The intermediate imidazole *N*-oxides **11** were prepared by AcOH-catalyzed condensation of formaldimines with diacetyl monoxime, as described [36]. Sulphuration of the latter imidazole *N*-oxides was carried out using cyclobutane-derived thioketone according to general report [42].

### 3.2. General Procedure for Synthesis of Cycloadducts ***16*** and ***17***

In a round-bottomed flask equipped with a stirring bar, an imidazole-2-thione **7** or **8** (1.2 mmol) was dissolved in dry THF (5 mL), the hydrazonoyl bromide **10** (1.0 mmol) was added followed by triethylamine (505 mg, 0.7 mL, 5.0 mmol), the flask was covered with a stopper, and the solution was stirred at ambient temperature until the starting hydrazonoyl bromide was fully consumed (TLC monitoring; typically 6 h for reactions of imidazole-thiones **7**, and 4 h for symmetric analogues **8**). The solvents were removed in vacuo, and the product was isolated via a standard column chromatography (CC) on silica gel (gradient petroleum ether to petroleum ether/CH_2_Cl_2_ 1:3 for hydrazonothioates **16**; gradient hexane to hexane/AcOEt 2:3 for spiro-products **17**), and recrystallized from a petroleum ether/dichloromethane mixture (in the case of products **16**).

*1-Benzyl-4,5-dimethylimidazol-2-yl 2,2,2-trifluoro-N′-(4-tolyl)-ethanehydrazonothioate* (**16a**): colorless crystals, 218 mg (52%), mp 105–106 °C. ^1^H NMR (600 MHz, CDCl_3_) *δ* 1.98, 2.19, 2.32 (3s, 3H each), 5.29 (s, 2H), 6.93–6.95 (m, 2H), 7.12–7.17 (m, 4H), 7.27–7.33 (m, 3H), 11.37 (s_br_, 1H). ^13^C NMR (151 MHz, CDCl_3_) *δ* 9.6, 12.8, 20.9, 48.4, 113.2 (q, ^2^*J*_C-F_ = 37.6 Hz), 114.2, 121.2 (q, ^1^*J*_C-F_ = 271.5 Hz, CF_3_), 126.2, 127.5, 127.9, 129.0, 129.90, 129.92, 132.1, 136.00, 136.01, 140.6. ^19^F NMR (565 MHz, CDCl_3_) *δ* −65.32 (s, CF_3_). ESI-MS (*m*/*z*) 419.5 (100, [M+H]^+^). IR (neat) *v* 1569, 1513, 1349, 1256, 1156, 1081 cm^−1^. Anal. Calcd for C_21_H_21_F_3_N_4_S (418.5): C 60.27, H 5.06, N 13.39, S 7.66; found: C 60.11, H 5.19, N 13.34, S 7.69.

*1-(3-Methoxybenzyl)-4,5-dimethylimidazol-2-yl 2,2,2-trifluoro-N′-(4-tolyl)-ethanehydrazonothioate* (**16b**): thick yellow oil, 247 mg (55%). ^1^H NMR (600 MHz, CDCl_3_) *δ* 1.99, 2.19, 2.32, 3.74 (4s, 3H each), 5.26 (s, 2H), 6.47 (m_c_, 1H), 6.52–6.54 (m, 1H), 6.81–6.83 (m, 1H), 7.12–7.17 (m, 4H), 7.22–7.25 (m, 1H), 11.36 (s_br_, 1H). ^13^C NMR (151 MHz, CDCl_3_) *δ* 9.6, 12.8, 20.8, 48.3, 55.3, 111.9, 113.19 (q, ^2^*J*_C-F_ = 37.6 Hz), 113.21, 114.2, 118.4, 121.1 (q, ^1^*J*_C-F_ = 271.6 Hz, CF_3_), 127.5, 129.8, 129.89, 130.08, 132.1, 136.0, 137.6, 140.6, 160.2. ^19^F NMR (565 MHz, CDCl_3_) *δ* −65.27 (s, CF_3_). ESI-MS (*m*/*z*) 449.4 (100, [M+H]^+^). IR (neat) *v* 1599, 1566, 1506, 1446, 1264, 1174, 1122, 1029 cm^−1^. Anal. Calcd for C_22_H_23_F_3_N_4_SO (448.5): C 58.92, H 5.17, N 12.49, S 7.15; found: C 58.95, H 5.17, N 12.41, S 7.34.

*1-Benzyl-4,5-dimethylimidazol-2-yl 2,2,2-trifluoro-N′-(4-metoxyphenyl)-ethanehydrazonothioate* (**16c**): beige crystals, 274 mg (63%), mp 90–92 °C. ^1^H NMR (600 MHz, CDCl_3_) *δ* 1.98, 2.18, 3.79 (3s, 3H each), 5.28 (s, 2H), 6.87–6.89 (m, 2H), 6.93–6.95 (m, 2H), 7.17–7.19 (m, 2H), 7.26–7.33 (m, 3H), 11.33 (s_br_, 1H). ^13^C NMR (151 MHz, CDCl_3_) *δ* 9.7, 12.9, 48.4, 55.8, 112.6 (q, ^2^*J*_C-F_ = 37.7 Hz), 114.8, 115.4, 121.2 (q, ^1^*J*_C-F_ = 271.4 Hz, CF_3_), 126.2, 127.4, 128.0, 129.0, 130.0, 135.9, 136.0, 136.8, 155.6. ^19^F NMR (565 MHz, CDCl_3_) *δ* −65.19 (s, CF_3_). ESI-MS (*m*/*z*) 435.3 (100, [M+H]^+^). IR (neat) *v* 1562, 1506, 1407, 1331, 1234, 1141, 1096, 1036, 969 cm^−1^. Anal. Calcd for C_21_H_21_F_3_N_4_SO (434.5): C 58.05, H 4.87, N 12.90, S 7.38; found: C 58.00, H 4.79, N 12.77, S 7.57.

*1-Benzyl-4,5-dimethylimidazol-2-yl 2,2,2-trifluoro-N′-phenyl-ethanehydrazonothioate* (**16d**): cream solid, 210 mg (52%), mp 89-91 °C. ^1^H NMR (600 MHz, CDCl_3_) *δ* 1.99, 2.20 (2s, 3H each), 5.29 (s, 2H), 6.93–6.95 (m, 2H), 7.00–7.02 (m, 1H), 7.24–7.27 (m, 2H), 7.28–7.34 (m, 5H), 11.45 (s_br_, 1H). ^13^C NMR (151 MHz, CDCl_3_) *δ* 9.7, 12.9, 48.4, 114.1 (q, ^2^*J*_C-F_ = 37.6 Hz), 114.3, 121.1 (q, ^1^*J*_C-F_ = 271.8 Hz, CF_3_), 122.6, 126.2, 127.6, 128.0, 129.1, 129.4, 129.8, 136.0, 136.1, 142.9. ^19^F NMR (565 MHz, CDCl_3_) *δ* −65.53 (s, CF_3_). ESI-MS (*m*/*z*) 405.3 (100, [M+H]^+^). IR (neat) *v* 1621, 1547, 1491, 1457, 1402, 1338, 1252, 1148, 1096, 1077 cm^−1^. Anal. Calcd for C_20_H_19_F_3_N_4_S (404.5): C 59.39, H 4.74, N 13.85, S 7.93; found: C 59.38, H 4.74, N 13.79, S 8.01.

*1-Benzyl-4,5-dimethylimidazol-2-yl 2,2,2-trifluoro-N′-(4-chlorophenyl)-ethanehydrazonothioate* (**16e**): colorless solid, 264 mg (60%), mp 114–116 °C (decomp.). ^1^H NMR (600 MHz, CDCl_3_) *δ* 1.99, 2.18 (2s, 3H each), 5.27 (s, 2H), 6.92–6.94 (m, 2H), 7.16–7.19 (m, 2H), 7.27–7.33 (m, 5H), 11.57 (s_br_, 1H). ^13^C NMR (151 MHz, CDCl_3_) *δ* 9.7, 12.9, 48.5, 115.0 (q, ^2^*J*_C-F_ = 37.7 Hz), 115.5, 120.9 (q, ^1^*J*_C-F_ = 271.9 Hz, CF_3_), 126.2, 127.5, 127.6, 128.0, 129.1, 129.4, 129.6, 135.9, 136.1, 141.6. ^19^F NMR (565 MHz, CDCl_3_) *δ* −65.76 (s, CF_3_). (−)-ESI-MS (*m*/*z*) 438.9 (35, [M{^37^Cl}-H]^−^), 437.0 (100, [M{^35^Cl}-H]^−^). IR (neat) *v* 1558, 1487, 1405, 1327, 1159, 1107, 973 cm^−1^. Anal. Calcd for C_20_H_18_ClF_3_N_4_S (438.9): C 54.73, H 4.13, N 12.77, S 7.30; found: C 54.72, H 4.08, N 12.80, S 7.44.

*1-Benzyl-4,5-dimethylimidazol-2-yl 2,2,2-trifluoro-N′-(4-bromophenyl)-ethanehydrazonothioate* (**16f**): beige solid, 261 mg (54%), mp 108–110 °C. ^1^H NMR (600 MHz, CDCl_3_) *δ* 1.99, 2.17 (2s, 3H each), 5.27 (s, 2H), 6.92–6.94 (m, 2H), 7.11–7.13 (m, 2H), 7.26–7.33 (m, 3H), 7.40–7.43 (m, 2H), 11.58 (s_br_, 1H). ^13^C NMR (151 MHz, CDCl_3_) *δ* 9.7, 12.9, 48.5, 114.9, 115.2 (q, ^2^*J*_C-F_ = 37.8 Hz), 115.9, 120.9 (q, ^1^*J*_C-F_ = 271.8 Hz, CF_3_), 126.2, 127.7, 128.0, 129.1, 129.6, 132.3, 135.9, 136.1, 142.1. ^19^F NMR (565 MHz, CDCl_3_) *δ* −65.80 (s, CF_3_). (−)-ESI-MS (*m*/*z*) 483.1 (97, [M{^81^Br}-H]^−^), 482.1 (100, [M{^79^Br}-H]^−^). IR (neat) *v* 1558, 1484, 1406, 1327, 1141, 1159, 1100, 973 cm^−1^. Anal. Calcd for C_20_H_18_BrF_3_N_4_S (483.4): C 49.70, H 3.75, N 11.59, S 6.63; found: C 49.78, H 3.85, N 11.65, S 6.86.

*1-Benzyl-4,5-dimethylimidazol-2-yl 2,2,2-trifluoro-N′-(4-benzoyloxyphenyl)-ethanehydrazonothioate* (**16g**): beige crystals, 319 mg (61%), mp 125–128 °C (decomp.). ^1^H NMR (600 MHz, CDCl_3_) *δ* 1.99, 2.19 (2s, 3H each), 5.29 (s, 2H), 6.93–6.94 (m, 2H), 7.17–7.19 (m, 2H), 7.27–7.33 (m, 5H), 7.50–7.53 (m, 2H), 7.62–7.65 (m, 1H), 8.20–8.22 (m, 2H), 11.53 (s_br_, 1H). ^13^C NMR (151 MHz, CDCl_3_) *δ* 9.7, 12.9, 48.5, 114.5 (q, ^2^*J*_C-F_ = 37.4 Hz), 115.0, 121.0 (q, ^1^*J*_C-F_ = 271.9 Hz, CF_3_), 122.7, 126.2, 127.7, 128.0, 128.7, 129.1, 129.7, 129.8, 130.3, 133.7, 135.9, 136.1, 140.8, 146.2, 165.6. ^19^F NMR (565 MHz, CDCl_3_) *δ* −65.62 (s, CF_3_). ESI-MS (*m*/*z*) 525.3 (100, [M+H]^+^). IR (neat) *v* 1737, 1558, 1502, 1331, 1245, 1193, 1144, 1111, 1062, 1025, 977 cm^−1^. Anal. Calcd for C_27_H_23_F_3_N_4_SO_2_ (524.6): C 61.82, H 4.42, N 10.68, S 6.11; found: C 61.60, H 4.27, N 10.41, S 6.09.

*1-Benzyl-4,5-dimethylimidazol-2-yl 2,2,2-trifluoro-N′-(4-cyanophenyl)-ethanehydrazonothioate* (**16h**): beige crystals, 218 mg (51%), mp 118–120 °C (decomp.). ^1^H NMR (600 MHz, CDCl_3_) *δ* 2.00, 2.18 (2s, 3H each), 5.27 (s, 2H), 6.92–6.93 (m, 2H), 7.28–7.34 (m, 5H), 7.58–7.61 (m, 2H), 12.02 (s_br_, 1H). ^13^C NMR (151 MHz, CDCl_3_) *δ* 9.7, 12.9, 48.5, 105.1, 114.5, 118.4 (q, ^2^*J*_C-F_ = 37.8 Hz), 119.5, 120.5 (q, ^1^*J*_C-F_ = 272.7 Hz, CF_3_), 126.1, 127.9, 128.1, 129.08, 129.13, 133.8, 135.7, 136.2, 146.4. ^19^F NMR (565 MHz, CDCl_3_) *δ* −66.39 (s, CF_3_). (−)-ESI-MS (*m*/*z*) 428.0 (100, [M-H]^−^). IR (neat) *v* 2215, 1602, 1551, 1502, 1413, 1338, 1297, 1249, 1129, 977 cm^−1^. Anal. Calcd for C_21_H_18_F_3_N_5_S (429.5): C 58.73, H 4.22, N 16.31, S 7.47; found: C 58.71, H 4.27, N 16.27, S 7.62.

*1-Benzyl-4,5-dimethylimidazol-2-yl 2,2,2-trifluoro-N′-(4-nitrophenyl)-ethanehydrazonothioate* (**16i**): yellow crystals, 260 mg (58%), mp 108–111 °C (decomp.). ^1^H NMR (600 MHz, CDCl_3_) *δ* 2.01, 2.19 (2s, 3H each), 5.28 (s, 2H), 6.93–6.94 (m, 2H), 7.28–7.34 (m, 5H), 8.20–8.24 (m, 2H), 12.22 (s_br_, 1H). ^13^C NMR (151 MHz, CDCl_3_) *δ* 9.7, 12.9, 48.5, 113.8, 119.4 (q, ^2^*J*_C-F_ = 37.9 Hz), 120.5 (q, ^1^*J*_C-F_ = 272.9 Hz, CF_3_), 125.9, 126.1, 128.0, 128.2, 129.9, 129.2, 135.6, 136.2, 142.6, 148.2. ^19^F NMR (565 MHz, CDCl_3_) *δ* −66.53 (s, CF_3_). ESI-MS (*m*/*z*) 450.3 (100, [M+H]^+^). IR (neat) *v* 1580, 1551, 1495, 1331, 1252, 1148, 1107 cm^−1^. Anal. Calcd for C_20_H_18_F_3_N_5_O_2_S (449.5): C 53.45, H 4.04, N 15.58, S 7.13; found: C 53.37, H 4.10, N 15.43, S 7.21.

*1′,3′-Dibenzyl-4′,5′-dimethyl-4-(4-tolyl)-2-trifluoromethylspiro [1,3,4-thiadiazole-5,2′-imidazole]* (**17a**): colorless solid, 376 mg (74%), mp 143–144 °C. ^1^H NMR (600 MHz, CDCl_3_) *δ* 2.06 (s, 6H), 2.16 (s, 3H), 5.08 (s, 4H), 6.55–6.57 (m, 2H), 6.80–6.82 (m, 2H), 7.11–7.14 (m, 4H), 7.19–7.21 (m, 6H). ^13^C NMR (151 MHz, CDCl_3_) *δ* 9.9, 20.6, 50.3, 117.5, 121.0 (q, ^1^*J*_C-F_ = 277.0 Hz, CF_3_), 124.6, 127.9, 128.3, 128.9, 129.4, 133.0, 133.1, 142.5, 142.9, 167.2 (q, ^2^*J*_C-F_ = 33.3 Hz). ^19^F NMR (565 MHz, CDCl_3_) *δ* −66.46 (s, CF_3_). ESI-MS (*m*/*z*) 509.3 (100, [M+H]^+^). IR (neat) *v* 1498, 1450, 1297, 1238, 1156, 1111, 1077, 991 cm^−1^. Anal. Calcd for C_28_H_27_F_3_N_4_S (508.6): C 66.12, H 5.35, N 11.02, S 6.30; found: C 66.21, H 5.32, N 10.88, S 6.43.

*1′-Benzyl-3′-(3-methoxybenzyl)-4′,5′-dimethyl-4-(4-tolyl)-2-trifluoromethylspiro [1,3,4-thiadiazole-5,2′-imidazole]* (**17b**): colorless solid, 290 mg (54%), mp 137–139 °C. ^1^H NMR (600 MHz, CDCl_3_) *δ* 2.08, 2.09, 2.18, 3.67 (4s, 3H each), 5.06, 5.10 (2s, 2H each), 6.56–6.59 (m, 2H), 6.71–6.76 (m, 3H), 6.81–6.84 (m, 2H), 7.13–7.15 (m, 3H), 7.20–7.23 (m, 3H). ^13^C NMR (151 MHz, CDCl_3_) *δ* 9.96, 9.97, 20.7, 50.44, 50.47, 55.5, 113.6, 114.3, 117.6, 120.2, 121.0 (q, ^1^*J*_C-F_ = 276.9 Hz, CF_3_), 124.6, 124.7, 128.0, 128.4, 129.0, 129.5, 129.9, 133.1, 133.2, 134.7, 142.7, 143.1, 160.1, 167.3 (q, ^2^*J*_C-F_ = 33.2 Hz). ^19^F NMR (565 MHz, CDCl_3_) *δ* −66.56 (s, CF_3_). IR (neat) *v* 1498, 1439, 1298, 1286, 1238, 1166, 1111, 1077 cm^−1^. HRMS (ESI-TOF) *m/z*: [M+H]^+^ calcd for C_29_H_30_F_3_N_4_OS 539.2092, found 539.2098.

*1′,3′-Bis(3-methoxybenzyl)-4′,5′-dimethyl-4-(4-tolyl)-2-trifluoromethylspiro [1,3,4-thiadiazole-5,2′-imidazole]* (**17c**): colorless solid, 284 mg (50%), mp 124–126 °C. ^1^H NMR (600 MHz, CDCl_3_) *δ* 2.09 (s, 6H), 2.18 (s, 3H), 3.66 (s, 6H), 5.05 (s, 4H), 6.56–6.58 (m, 2H), 6.71–6.75 (m, 6H), 6.82–6.84 (m, 2H), 7.12–7.15 (m, 2H). ^13^C NMR (151 MHz, CDCl_3_) *δ* 9.9, 20.7, 50.4, 55.5, 113.6, 114.4, 117.5, 120.1, 121.1 (q, ^1^*J*_C-F_ = 276.9 Hz, CF_3_), 124.7, 129.5, 129.9, 133.0, 134.6, 142.7, 143.0, 160.1, 167.1 (q, ^2^*J*_C-F_ = 32.1 Hz). ^19^F NMR (565 MHz, CDCl_3_) *δ* −66.54 (s, CF_3_). IR (neat) *v* 1603, 1491, 1439, 1260, 1159, 1115, 980 cm^−1^. HRMS (ESI-TOF) *m/z*: [M+H]^+^ calcd for C_30_H_32_F_3_N_4_O_2_S 569.2198, found 569.2198.

*1′,3′-Dibenzyl-4′,5′-dimethyl-4-(4-methoxyphenyl)-2-trifluoromethylspiro [1,3,4-thiadiazole-5,2′-imidazole]* (**17d**): pale yellow solid, 225 mg (43%), mp 49–51 °C. ^1^H NMR (600 MHz, CDCl_3_) *δ* 2.10 (s, 6H), 3.66 (s, 3H), 5.09 (s, 4H), 6.52–6.57 (m, 4H), 7.10–7.13 (m, 4H), 7.20–7.23 (m, 6H). ^13^C NMR (151 MHz, CDCl_3_) *δ* 9.9, 50.3, 56.7, 114.3, 119.7, 121.0 (q, ^1^*J*_C-F_ = 276.9 Hz, CF_3_), 124.4, 127.9, 128.4, 129.0, 133.3, 138.3, 143.3, 156.3, 167.8 (q, ^2^*J*_C-F_ = 32.5 Hz). ^19^F NMR (565 MHz, CDCl_3_) *δ* −66.46 (s, CF_3_). IR (neat) *v* 1498, 1443, 1290, 1230, 1167, 1115, 1073, 1028, 991 cm^−1^. HRMS (ESI-TOF) *m/z*: [M+H]^+^ calcd for C_28_H_28_F_3_N_4_OS 525.1936, found 525.1938.

*1′,3′-Dibenzyl-4′,5′-dimethyl-4-phenyl-2-trifluoromethylspiro [1,3,4-thiadiazole-5,2′-imidazole]* (**17e**): pale yellow solid, 257 mg (52%), mp 57–59 °C. ^1^H NMR (600 MHz, CDCl_3_) *δ* 2.10 (s, 6H), 5.11 (s, 4H), 6.65–6.67 (m, 2H), 6.84–6.86 (m, 1H), 7.00–7.03 (m, 2H), 7.12–7.15 (m, 4H), 7.19–7.21 (m, 6H). ^13^C NMR (151 MHz, CDCl_3_) *δ* 10.0, 50.5, 117.2, 121.0 (q, ^1^*J*_C-F_ = 277.0 Hz, CF_3_), 123.3, 124.9, 127.1, 128.0, 128.5, 128.9, 129.0, 133.0, 142.8, 144.9, 167.4 (q, ^2^*J*_C-F_ = 32.4 Hz). ^19^F NMR (565 MHz, CDCl_3_) *δ* −66.57 (s, CF_3_). IR (neat) *v* 1595, 1491, 1454, 1290, 1230, 1167, 1118, 1059, 988 cm^−1^. HRMS (ESI-TOF) *m/z*: [M+H]^+^ calcd for C_27_H_26_F_3_N_4_S 495.1830, found 495.1835.

*4-(4-Chlorophenyl)-1′,3′-dibenzyl-4′,5′-dimethyl-2-trifluoromethylspiro [1,3,4-thiadiazole-5,2′-imidazole]* (**17f**): colorless solid, 391 mg (74%), mp 63–65 °C. ^1^H NMR (600 MHz, CDCl_3_) *δ* 2.17 (s, 6H), 5.10 (s, 4H), 6.46–6.48 (m, 2H), 6.86–6.88 (m, 2H), 7.09–7.10 (m, 4H), 7.18–7.24 (m, 6H). ^13^C NMR (151 MHz, CDCl_3_) *δ* 10.0, 50.4, 118.2, 120.9 (q, ^1^*J*_C-F_ = 277.2 Hz, CF_3_), 124.9, 128.0, 128.2, 128.56, 128.64, 129.0, 132.8, 142.3, 143.4, 168.2 (q, ^2^*J*_C-F_ = 32.4 Hz). ^19^F NMR (565 MHz, CDCl_3_) *δ* −66.58 (s, CF_3_). IR (neat) *v* 1484, 1454,1286, 1234, 1170, 1118,1006, 988 cm^−1^. HRMS (ESI-TOF) *m/z*: [M+H]^+^ calcd for C_27_H_25_ClF_3_N_4_S 529.1441, found 529.1447.

*4-(4-Bromophenyl)-1′,3′-dibenzyl-4′,5′-dimethyl-2-trifluoromethylspiro [1,3,4-thiadiazole-5,2′-imidazole]* (**17g**): colorless solid, 400 mg (70%), mp 69–71 °C. ^1^H NMR (600 MHz, CDCl_3_) *δ* 2.17 (s, 6H), 5.09 (s, 4H), 6.40–6.42 (m, 2H), 7.00–7.02 (m, 2H), 7.08–7.10 (m, 4H), 7.18–7.24 (m, 6H). ^13^C NMR (151 MHz, CDCl_3_) *δ* 10.0, 50.4, 115.8, 118.5, 120.9 (q, ^1^*J*_C-F_ = 277.1 Hz, CF_3_), 125.0, 128.0, 128.6, 129.0, 131.5, 132.7, 142.1, 143.9, 168.2 (q, ^2^*J*_C-F_ = 32.5 Hz). ^19^F NMR (565 MHz, CDCl_3_) *δ* −66.57 (s, CF_3_). IR (neat) *v* 1484, 1454, 1286, 1234, 1170, 1118, 1066, 1006, 987 cm^−1^. HRMS (ESI-TOF) *m/z*: [M+H]^+^ calcd for C_27_H_25_BrF_3_N_4_S 573.0935, found 573.0941.

*4-(4-Benzoyloxyphenyl)-1′,3′-dibenzyl-4′,5′-dimethyl-2-trifluoromethylspiro [1,3,4-thiadiazole-5,2′-imidazole]* (**17h**): colorless solid, 331 mg (54%), mp 149–150 °C. ^1^H NMR (600 MHz, CDCl_3_) *δ* 2.15 (s, 6H), 5.14 (s, 4H), 6.64–6.67 (m, 2H), 6.86–6.89 (m, 2H), 7.14–7.17 (m, 4H), 7.23–7.25 (m, 6H), 7.49–7.51 (m, 2H), 7.61–7.64 (m, 1H), 8.15–8.17 (m, 2H). ^13^C NMR (151 MHz, CDCl_3_) *δ* 10.0, 50.6, 117.9, 121.0 (q, ^1^*J*_C-F_ = 277.1 Hz, CF_3_), 122.0, 124.9, 128.1, 128.67, 128.70, 129.1, 129.7, 130.2, 132.9, 133.7, 142.7, 142.8, 146.7, 165.0, 167.5 (q, ^2^*J*_C-F_ = 32.6 Hz). ^19^F NMR (565 MHz, CDCl_3_) *δ* −66.61 (s, CF_3_). IR (neat) *v* 1737, 1498, 1454, 1356, 1282, 1200, 1192, 1107, 1059, 1006, 988 cm^−1^. HRMS (ESI-TOF) *m/z*: [M+H]^+^ calcd for C_34_H_30_F_3_N_4_O_2_S 615.2042, found 615.2037.

*4-(4-Cyanophenyl)-1′,3′-dibenzyl-4′,5′-dimethyl-2-trifluoromethylspiro [1,3,4-thiadiazole-5,2′-imidazole]* (**17i**): pale yellow solid, 312 mg (60%), mp 67–69 °C. ^1^H NMR (600 MHz, CDCl_3_) *δ* 2.27 (s, 6H), 5.08, 5.14 (2s_br_, 2H each), 6.45–6.48 (m, 2H), 7.05–7.09 (m, 6H), 7.13–7.20 (m, 6H). ^13^C NMR (151 MHz, CDCl_3_) *δ* 10.0, 50.5, 105.1, 116.1, 119.1, 120.8 (q, ^1^*J*_C-F_ = 277.4 Hz, CF_3_), 125.5, 128.1, 128.8, 129.1, 132.3, 132.5, 141.1, 148.1, 168.9 (q, ^2^*J*_C-F_ = 32.5 Hz). ^19^F NMR (565 MHz, CDCl_3_) *δ* −66.67 (s, CF_3_). IR (neat) *v* 2222, 1603, 1498, 1454, 1238, 1170, 1170, 1118, 1080, 1010, 988 cm^−1^. HRMS (ESI-TOF) *m/z*: [M+H]^+^ calcd for C_28_H_25_F_3_N_5_S 520.1783, found 520.1787.

*1′,3′-Dibenzyl-4′,5′-dimethyl-4-(4-nitrophenyl)-2-trifluoromethylspiro [1,3,4-thiadiazole-5,2′-imidazole]* (**17j**): light red solid, 216 mg (40%), mp 53–55 °C. ^1^H NMR (600 MHz, CDCl_3_) *δ* 2.30 (s, 6H), 5.08, 5.16 (2s_br_, 2H each), 6.44–6.48 (m, 2H), 7.06–7.09 (m, 4H), 7.11–7.16 (m, 6H), 7.66–7.68 (m, 2H). ^13^C NMR (151 MHz, CDCl_3_) *δ* 10.0, 50.6, 115.3, 120.8 (q, ^1^*J*_C-F_ = 277.5 Hz, CF_3_), 124.4, 125.6, 128.0, 128.8, 129.1, 132.2, 140.9, 142.1, 149.7, 169.3 (q, ^2^*J*_C-F_ = 32.6 Hz). ^19^F NMR (565 MHz, CDCl_3_) *δ* −66.67 (s, CF_3_). IR (neat) *v* 1592, 1495, 1454, 1334, 1271, 1238, 1170, 1107 cm^−1^. HRMS (ESI-TOF) *m/z*: [M+H]^+^ calcd for C_27_H_25_F_3_N_5_O_2_S 540.1681, found 540.1691.

## 4. Conclusions

In summary, two series of lepidiline-inspired imidazole-2(3*H*)-thiones were examined in reactions with in situ-generated trifluoromethylated nitrile imines. Enolizable imidazole-2-thiones solely provided the respective hydrazonothioates formed through nucleophilic *S*-addition onto the 1,3-dipole, whereas the *N*,*N*-disubstituted non-enolizable analogues afforded the corresponding (3+2)-cycloadducts in high yield. The latter products belong to a hitherto very little-known spiro [1,3,4-thiadiazole-5,2′-imidazole] family [52] and are the first examples of non-ionic spiro-analogues of naturally occurring lepidiline alkaloids as well as new fluorinated imidazole-thiadiazole hybrids of potential interest in medicine, agrochemistry, and material sciences [53,54,55]. The present work further expands the scope of products available by trapping of CF_3_-nitrile imines with thiocarbonyl substrates and reveals absorptions of the *C*-(CF_3_) atom in the ^13^C NMR spectrum as a useful probe for the differentiation of the open-chain, monocyclic, and spiro-cyclic products in the series. Furthermore, the presented results nicely supplement a more recent study on (chemo)selectivity problems in reactions of enolizable azaheterocyclic thiones [56,57].

## Data Availability

All the electronic experimental data and samples of new materials are available from the authors.

## Supplementary Materials

The following supporting information can be downloaded at https://www.mdpi.com/article/10.3390/molecules30193851/s1: Supplementary File S1. Copies of ^1^H, ^13^C, and ^19^F NMR spectra of all new compounds.
