# Supplementary material for: Lepidiline-Derived Imidazole-2(3H)-Thiones: (3+2)-Cycloadditions vs. Nucleophilic Additions in Reactions with Fluorinated Nitrile Imines
Source: Molecules. 2025 Sep 23;30(19):3851. doi: 10.3390/molecules30193851 (PMC12526023; doi:10.3390/molecules30193851)
Supplement: Supplementary file 1 [file molecules-30-03851-s001.zip › molecules-3840165-supplementary.pdf]

Supporting Information
for

Lepidiline-Derived Imidazole-2(3*H*)-thiones: (3+2)-Cycloadditions *vs*. Nucleophilic Additions in Reactions with Fluorinated Nitrile Imines

Wiktor K. Poper, Kamil Świątek, Katarzyna Urbaniak, Barbara Olszewska and Marcin Jasiński*

Department of Organic and Applied Chemistry, Faculty of Chemistry, University of Lodz, 91403 Łódź, Poland

**e-mail: mjasinski@uni.lodz.pl*

Content:

Synthesis of imidazole-2-thiones **7** and **8** S2-S3

Copies of ^{1}H, ^{13}C, and ^{19}F NMR spectra for new compounds S4-S24

Synthesis of imidazole-2-thiones 7 and 8

Synthesis of imidazole-2-thiones **7a**-**7b**: A mixture of benzylamine **12a** or **12b** (10.0 mmol) and aqueous formaldehyde (37%, 0.98 g, 12.0 mmol) in benzene (15 mL) was refluxed in a Dean−Stark apparatus for 2 h. The solvent was removed under reduced pressure to give trimer of formaldimine **13** (> 95% purity). To a solution of this crude trimeric formaldimine **13** in glacial acetic acid (20 mL) was added diacetyl monoxime (10.0 mmol), and the resulting mixture was stirred at room temperature overnight. Then, excess concentrated HCl was added (4 mL), the solvents were removed under reduced pressure, and the resulting product was dissolved in MeOH. After excess solid $NaHCO_3$ was added the stirring was continued for 2 h until the evolution of CO_2 ceased. The solvent was removed in vacuo, the residue was triturated with CH_2Cl_2, the precipitate salts were filtered off, the solvent was removed, and the residue was washed with a few portions of Et_2O to give crude imidazole *N*-oxide **11**. To a solution of this crude imidazole *N*-oxide in dry DCM (30.0 mL) was added dropwise a solution of 2,2,4,4-tetramethyl-3-thioxocyclo-butanone (1.9 g, 11.0 mmol) in DCM (15.0 mL), and the mixture was stirred at room temperature for 2 h. The solvent was removed *in vacuo*, the resulting was triturated with E_2O to give product **7**.

1-Benzyl-4,5-dimethylimidazole-2-thione (**7a**):[1] Colorless solid, 1.16 g (53%). ^{1}H NMR (600 MHz, $CDCl_3$) δ 1.91 (s, 3H), 2.08 (s, 3H), 5.29 (s, 2H), 7.24-7.27 (m, 3H), 7.30-7.33 (m, 2H), 11.07 (s_{br}, 1H).

1-(3-Methoxybenzyl)-4,5-dimethylimidazole-2-thione (**7b**): Colorless solid, 1.27 g (51%); mp 202-204 °C. ^{1}H NMR (600 MHz, $CDCl_3$) δ 1.92 (s, 3H), 2.07 (s 3H), 3.78 (s, 3H), 5.26 (s, 2H), 6.78-6.84 (m, 3H), 7.20-7.24 (m, 1H), 11.24 (s_{br}, 1H). ^{13}C NMR (151 MHz, $CDCl_3$) δ 9.2, 9.3, 48.0, 55.4, 112.9, 113.1, 119.4, 120.1, 121.9, 129.9, 138.1, 159.6, 160.1. IR (neat) v 3071, 2919, 1610, 1584, 1487, 1398, 1286, 1226, 1148, 1051 cm^{-1}. HRMS (ESI-TOF) *m/z*: $[M+H]^+$ calcd for $C_{13}H_{17}N_2OS$ 249.1062, found 249.1064.

Synthesis of imidazole-2-thiones **8a**-**8c**: To a solution of imidazole *N*-oxide **11** (2.0 mmol) in MeOH (5.0 mL) was added portion-wise an excess of freshly prepared suspension of Raney-nickel in MeOH. The resulting mixture was stirred at room temperature until the starting *N*-oxide was fully consumed (TLC monitoring; ca. 1.5 h). The solids were filtered off and the solvent was removed in vacuo to give spectroscopically pure imidazole **14**. To a deoxygenated solution of this imidazole **14** in MeCN (20 mL) was added benzyl chloride (380 mg, 3.0 mmol) or 3-methoxybenzyl chloride (470 mg, 3.0 mmol), and the resulting mixture was MW-irradiated at 110 °C until the starting imidazole was fully consumed (TLC monitoring; ca. 1 h). The solvent was removed under reduced pressure, and the crude product **15** was washed with several portions of dry Et_2O, dissolved in CH_2Cl_2 and precipitated by portion-wise addition of hexanes. The resulting imidazolium chloride **15** and elemental sulfur (4.0 mmol) in a pyridine/Et_3N mixture (1:1, 20.0 mL) were stirred at room temperature overnight. The solvents were removed in vacuo, and the obtained residue was purified by short chromatography column (using CH_2Cl_2 as an eluent) to give products **8a**-**8c**.

1,3-Dibenzyl-4,5-dimethylimidazole-2-thione (**8a**).[2] Colorless solid, 259 mg (42%). ^{1}H NMR (600 MHz, $CDCl_3$) δ 1.95 (s, 6H), 5.44 (s, 4H), 7.25-7.34 (m, 10H).

1-(3-Methoxybenzyl)-3-benzyl-4,5-dimethylimidazole-2-thione (**8b**).[2] Waxy solid, 480 mg (71%). ^{1}H NMR (600 MHz, $CDCl_3$) δ 1.95 (s, 3H), 1.96 (s, 3H), 3.78 (s, 3H), 5.41 (s, 2H), 5.44 (s, 2H), 6.79-6.81 (m, 1H), 6.83−6.87 (m, 2H), 7.22-7.25 (m, 1H), 7.26−7.33 (m, 5H).

---

[1] G. Mlostoń et al., *Helv. Chim. Acta* **1998**, 81, 1585−1595.
[2] G. Mlostoń et al., *J. Nat. Prod.* **2021**, *84*, 3071–3079.

1-(3-Methoxybenzyl)-3-benzyl-4,5-dimethylimidazole-2-thione (**8c**). Thick colorless oil, 486 mg (66%). ^{1}H NMR (600 MHz, $CDCl_3$) *δ* 1.96 (s, 6H), 3.78 (s, 6H), 5.41 (s, 4H), 6.79-6.81 (m, 2H), 6.83-6.86 (m, 4H), 7.21-7.24 (m, 2H). ^{13}C NMR (151 MHz, $CDCl_3$) *δ* 9.4, 48.9, 55.3, 112.7, 113.1, 119.3, 121.5, 129.8, 138.3, 160.0, 163.0. IR (neat) *v* 1599, 1584, 1491, 1454, 1398, 1341, 1260, 1226, 1148, 1040 cm^{-1}. HRMS (ESI-TOF) *m/z*: $[M+H]^+$ calcd for $C_{21}H_{24}N_2O_2S$ 369.1637, found 369.1642.

Copies of ^{1}H, ^{13}C, and ^{19}F NMR spectra for compounds 16 and 17

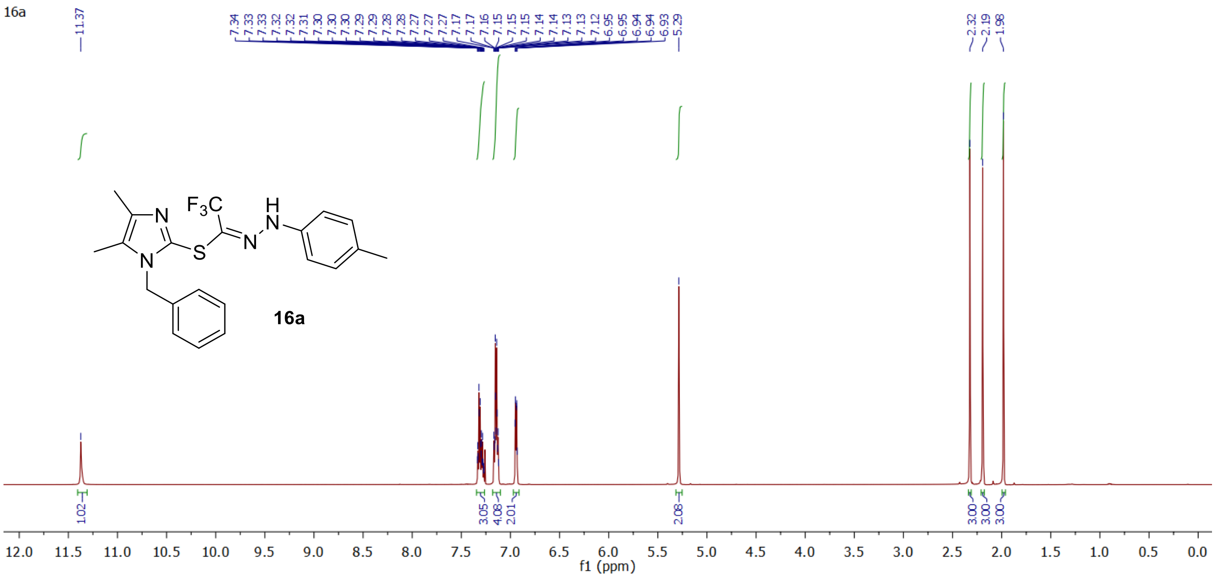

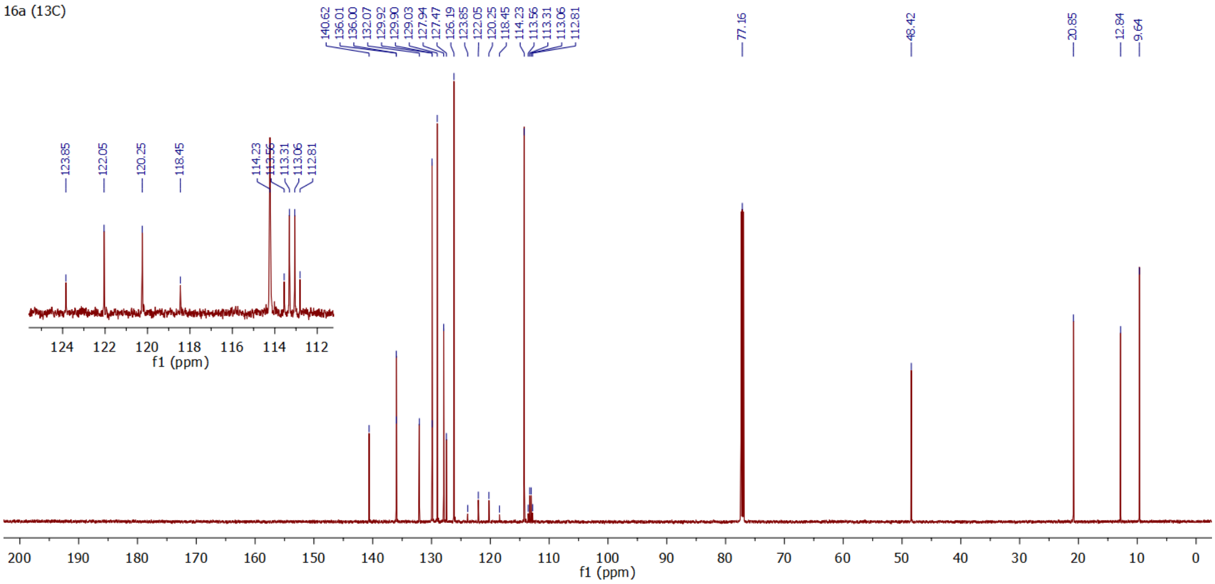

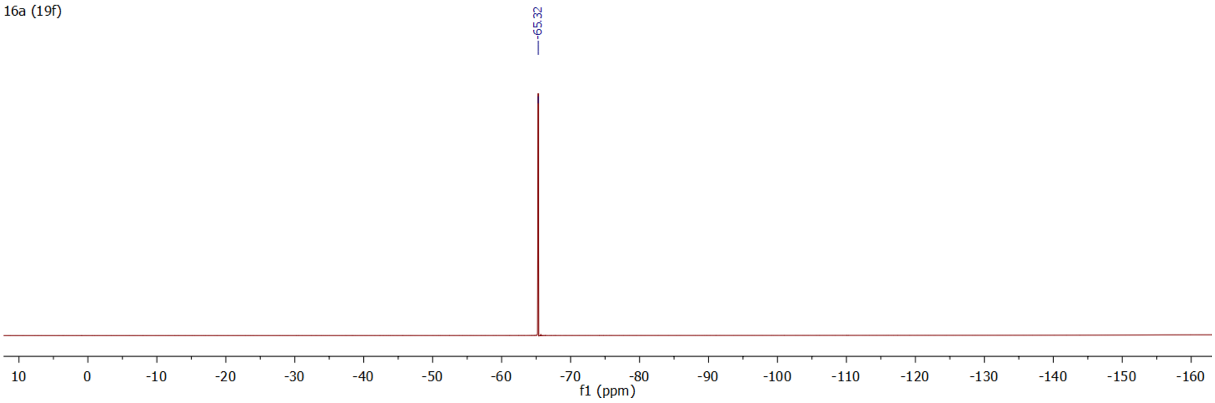

Figure S1. ^{1}H NMR (600 MHz, $CDCl_3$), ^{13}C NMR (151 MHz, $CDCl_3$) and ^{19}F NMR (565 MHz, $CDCl_3$) spectra for compound **16a**.

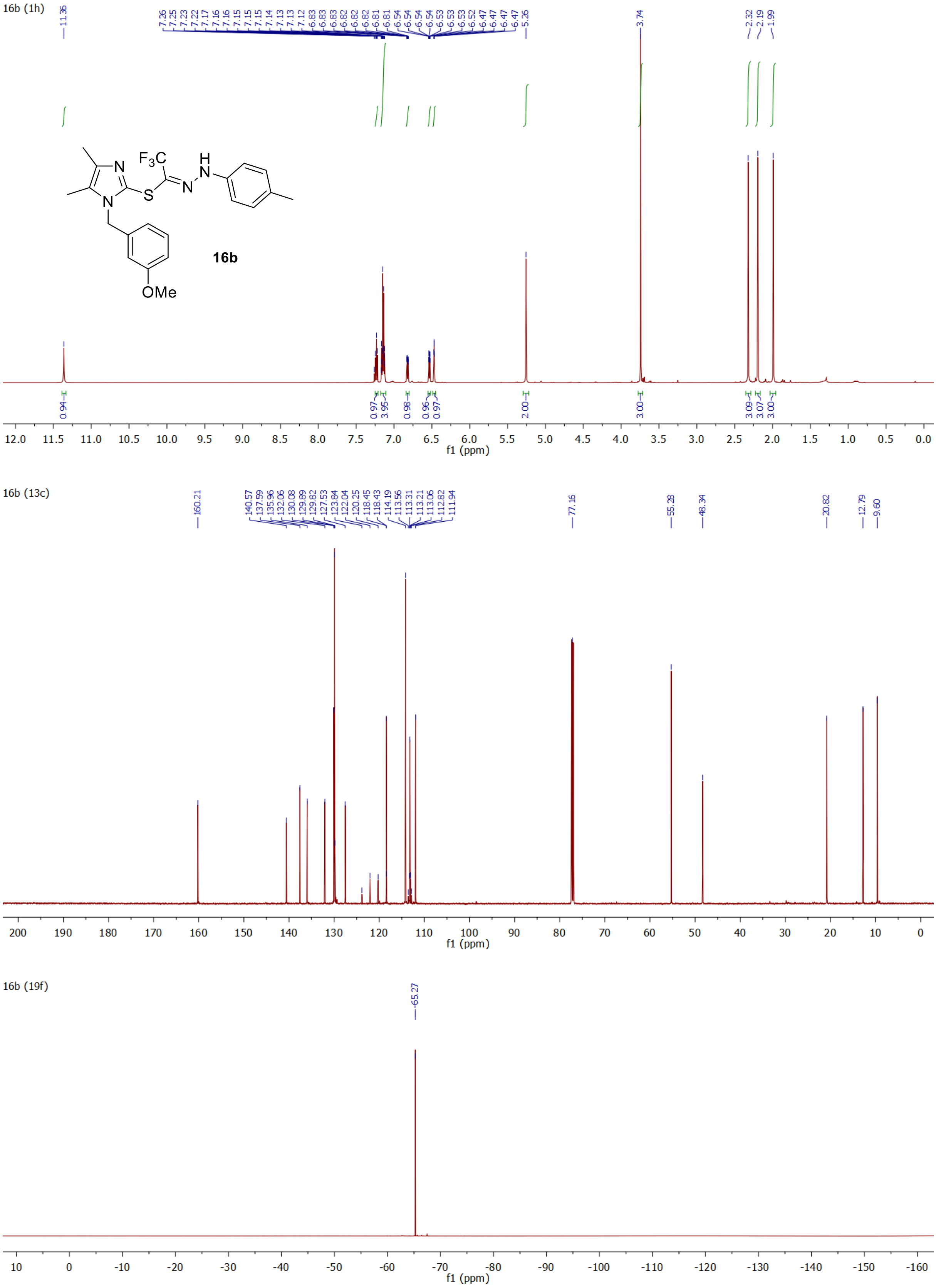

Figure S2. ^{1}H NMR (600 MHz, $CDCl_3$), ^{13}C NMR (151 MHz, $CDCl_3$) and ^{19}F NMR (565 MHz, $CDCl_3$) spectra for compound **16b**.

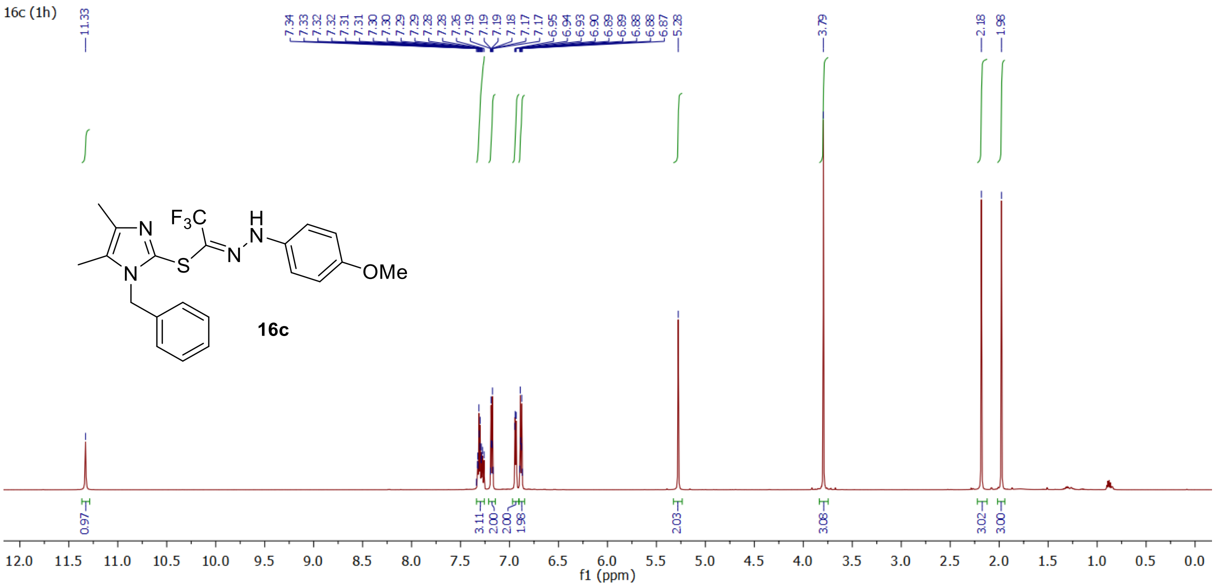

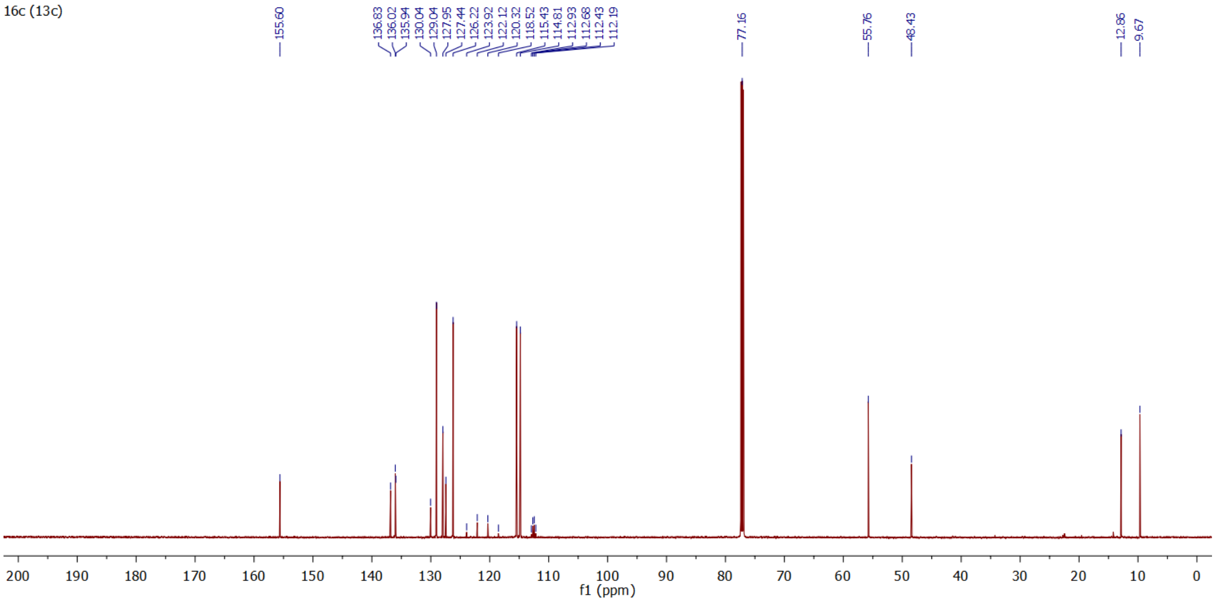

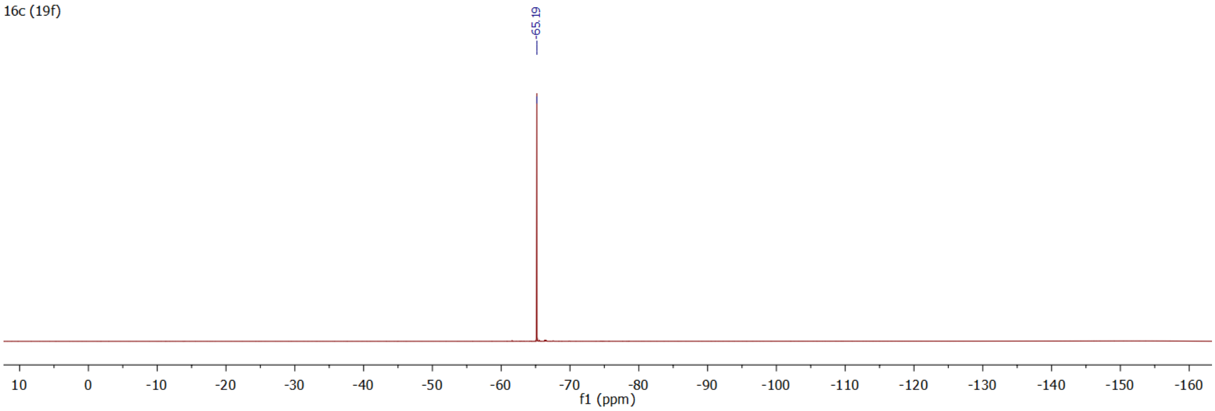

Figure S3. ^{1}H NMR (600 MHz, $CDCl_3$), ^{13}C NMR (151 MHz, $CDCl_3$) and ^{19}F NMR (565 MHz, $CDCl_3$) spectra for compound **16c**.

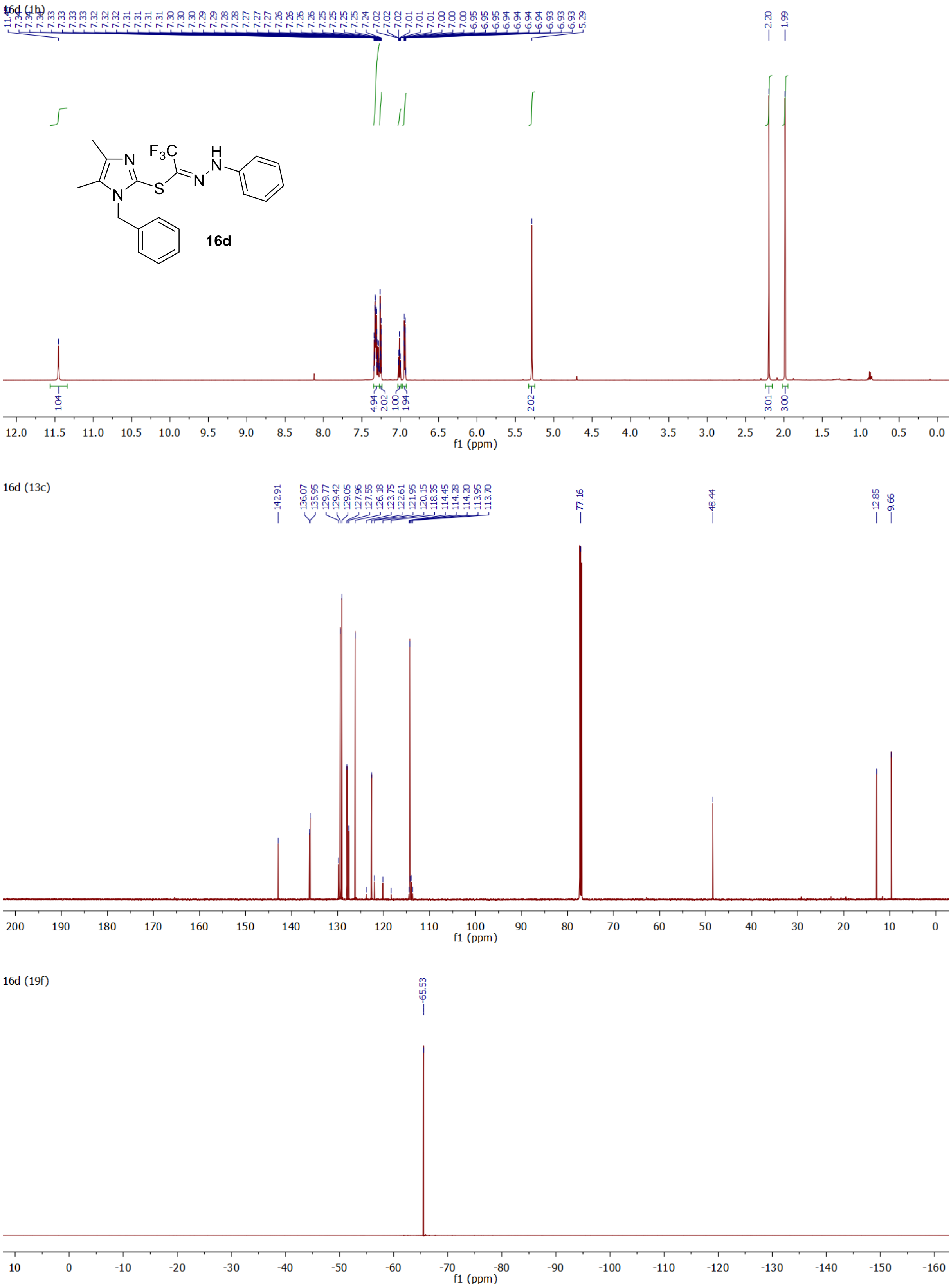

Figure S4. ^{1}H NMR (600 MHz, $CDCl_3$), ^{13}C NMR (151 MHz, $CDCl_3$) and ^{19}F NMR (565 MHz, $CDCl_3$) spectra for compound **16d**.

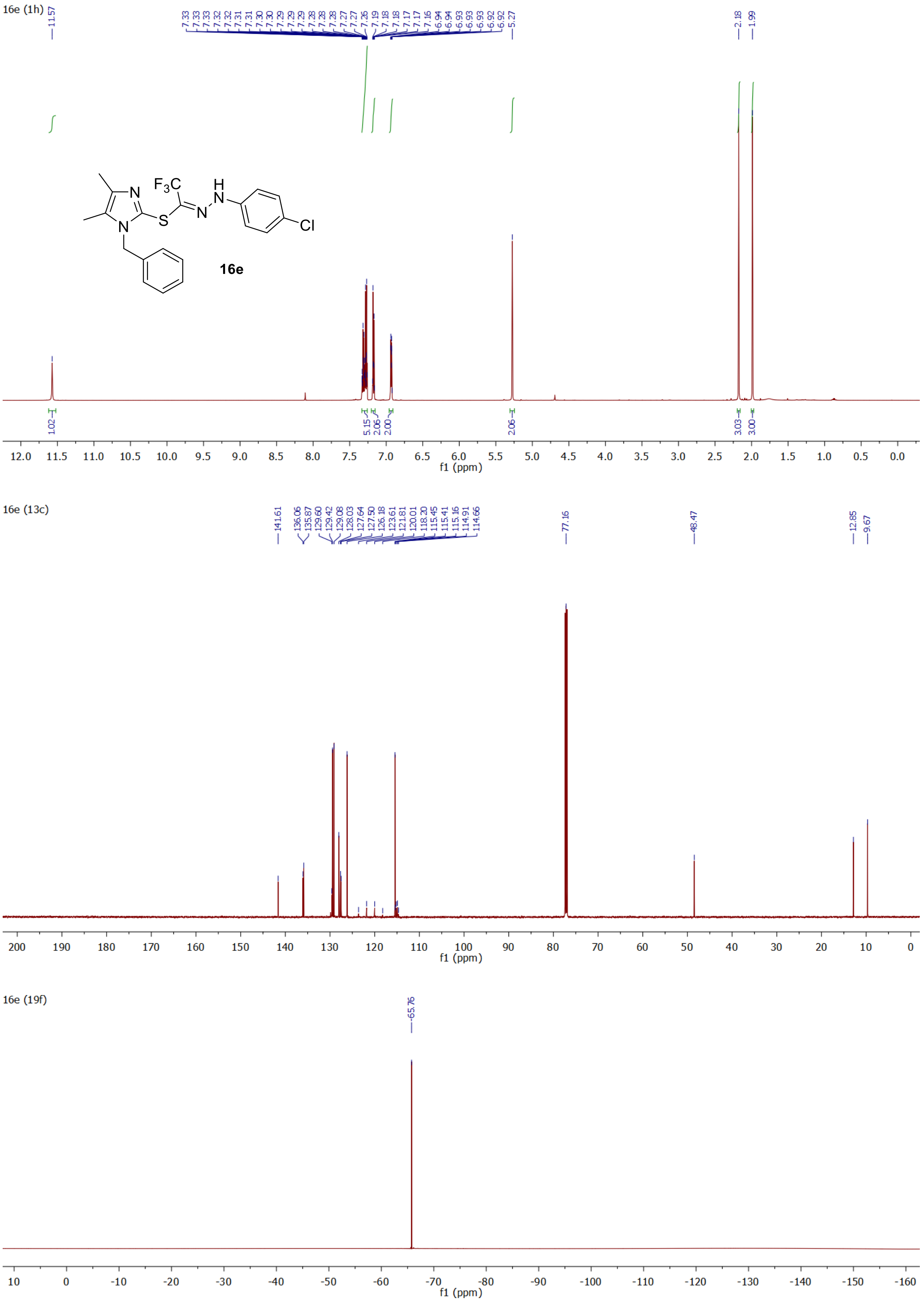

Figure S5. ^{1}H NMR (600 MHz, $CDCl_3$), ^{13}C NMR (151 MHz, $CDCl_3$) and ^{19}F NMR (565 MHz, $CDCl_3$) spectra for compound **16e**.

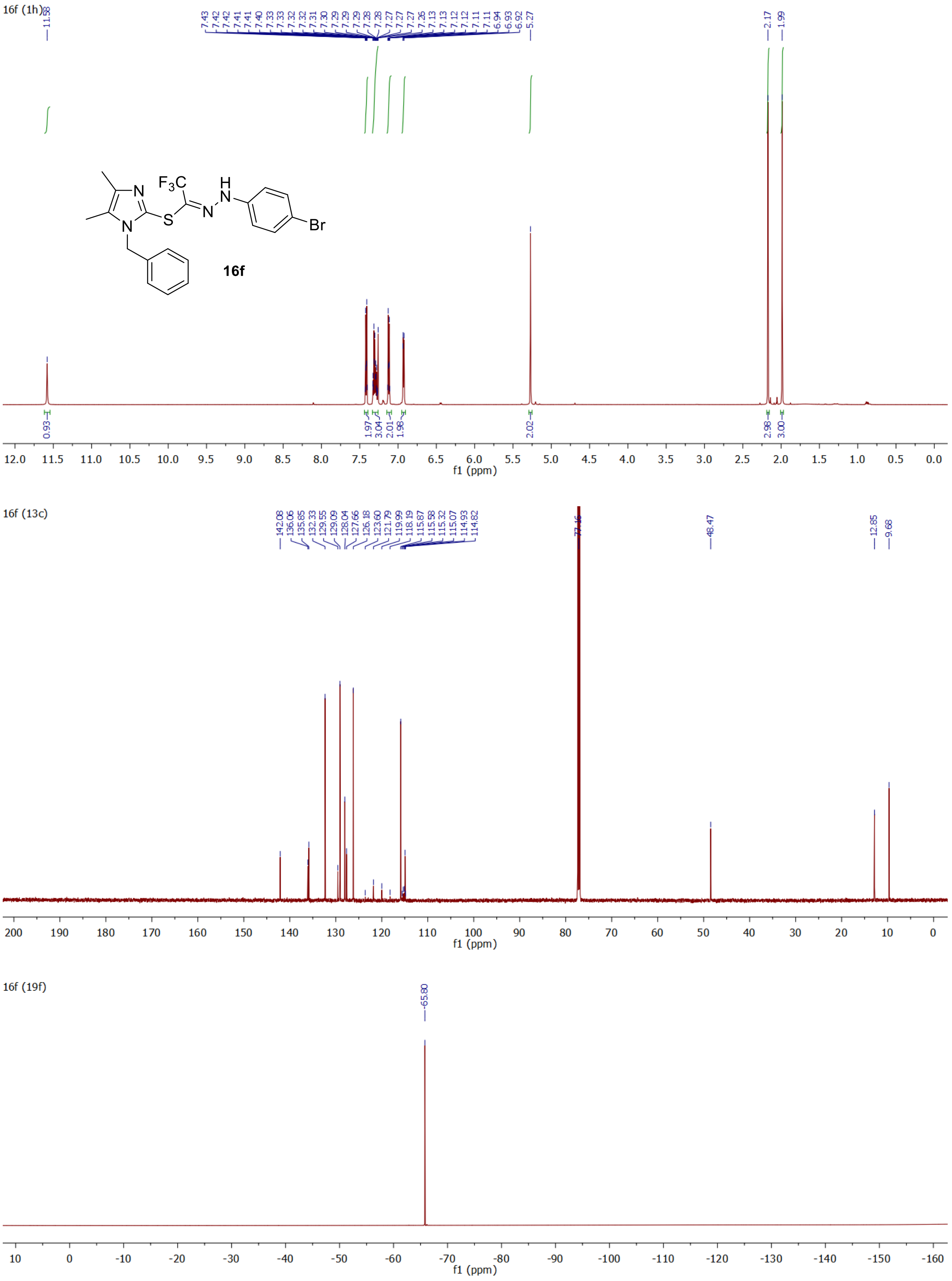

Figure S6. ^{1}H NMR (600 MHz, $CDCl_3$), ^{13}C NMR (151 MHz, $CDCl_3$) and ^{19}F NMR (565 MHz, $CDCl_3$) spectra for compound **16f**.

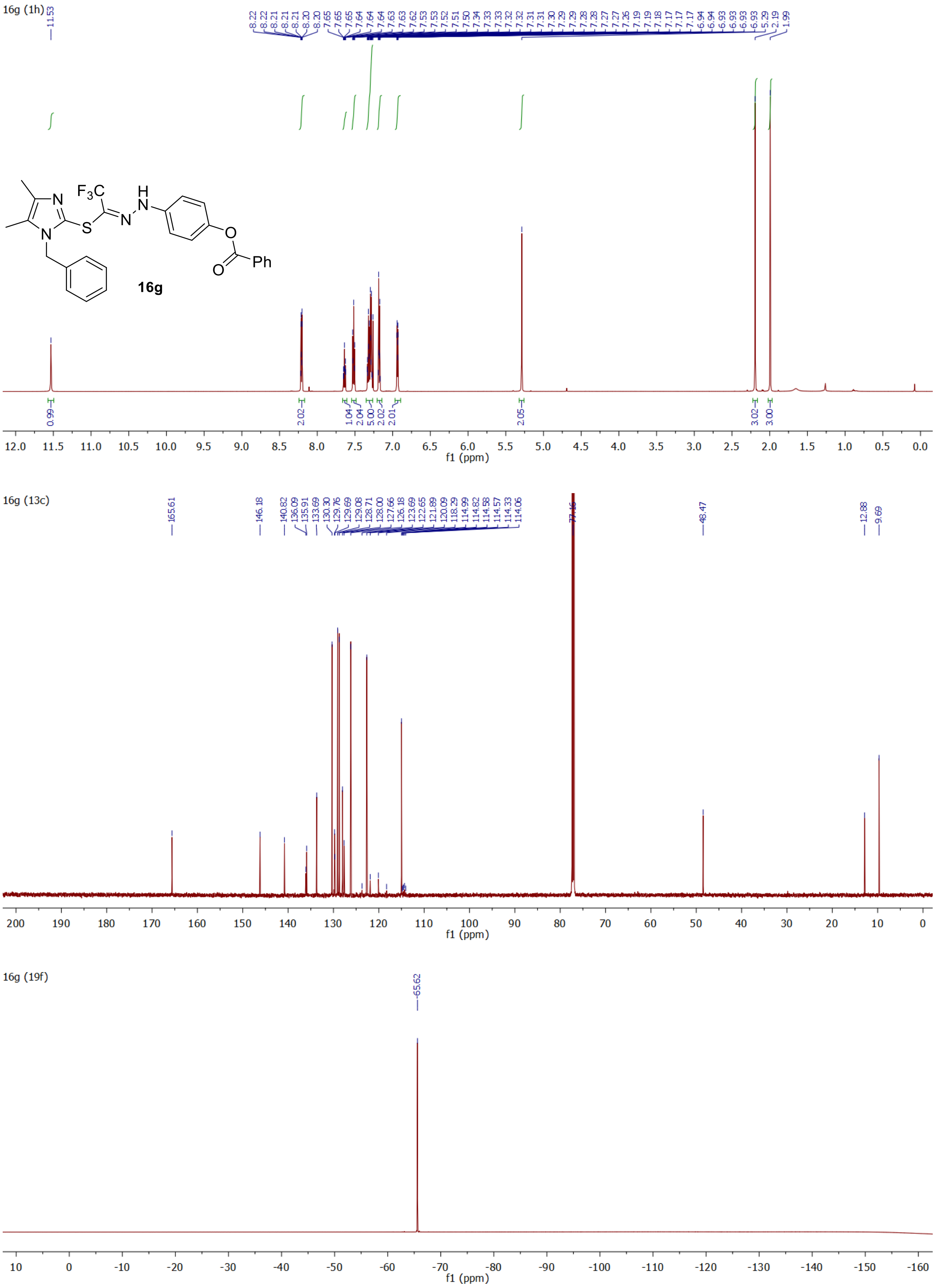

Figure S7. ^{1}H NMR (600 MHz, $CDCl_3$), ^{13}C NMR (151 MHz, $CDCl_3$) and ^{19}F NMR (565 MHz, $CDCl_3$) spectra for compound **16g**.

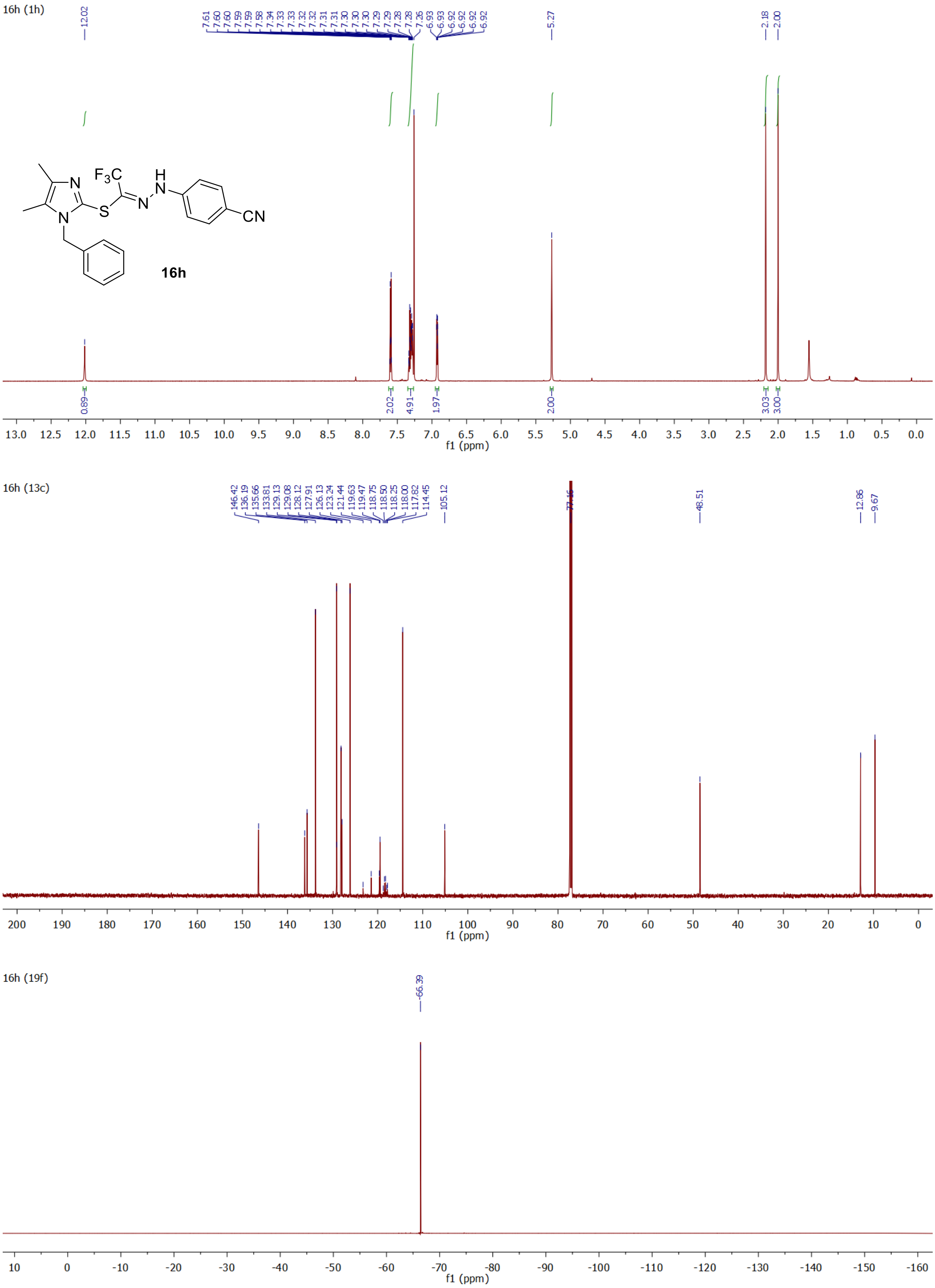

Figure S8. ^{1}H NMR (600 MHz, $CDCl_3$), ^{13}C NMR (151 MHz, $CDCl_3$) and ^{19}F NMR (565 MHz, $CDCl_3$) spectra for compound **16h**.

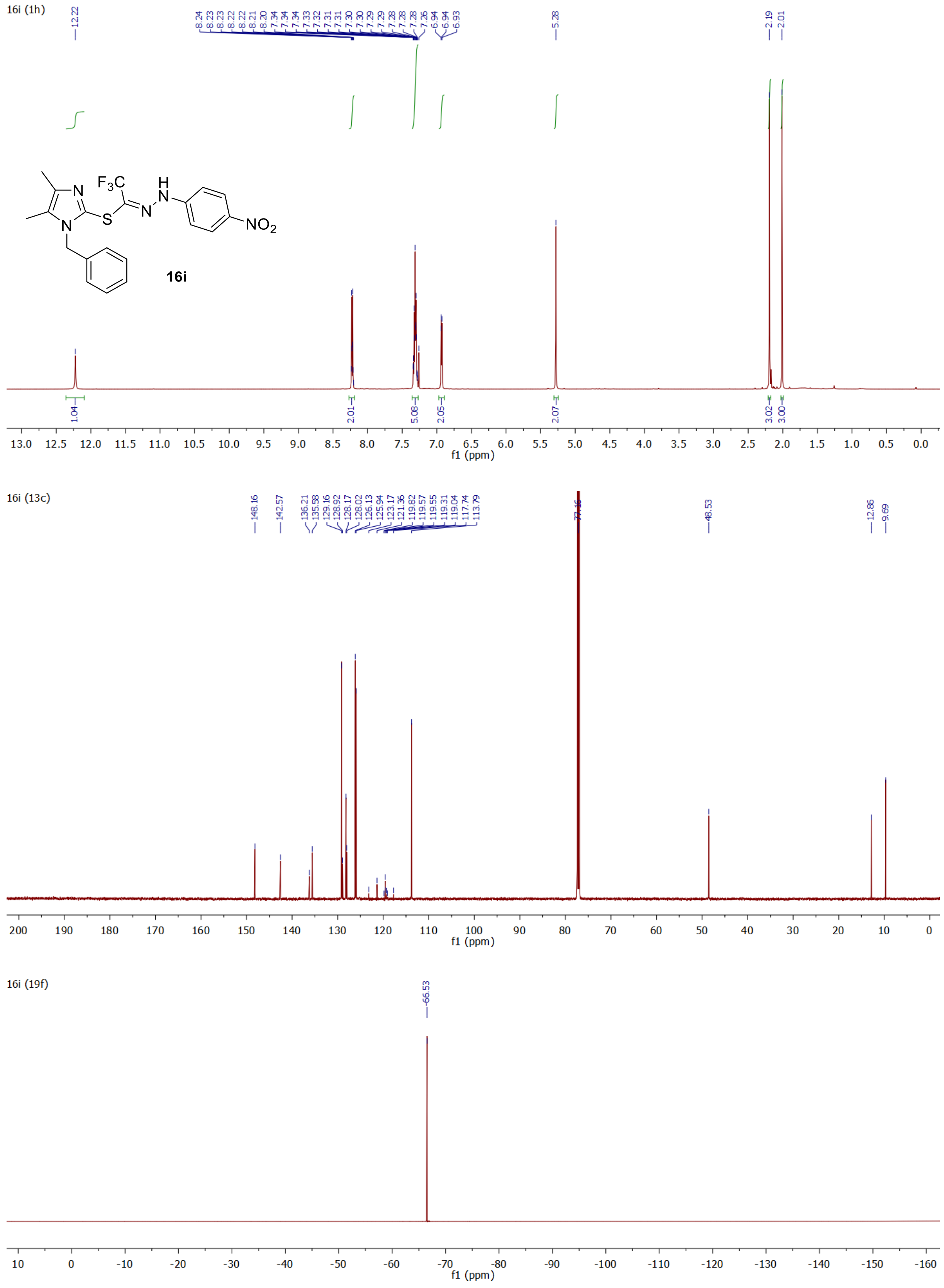

Figure S9. ^{1}H NMR (600 MHz, $CDCl_3$), ^{13}C NMR (151 MHz, $CDCl_3$) and ^{19}F NMR (565 MHz, $CDCl_3$) spectra for compound **16i**.

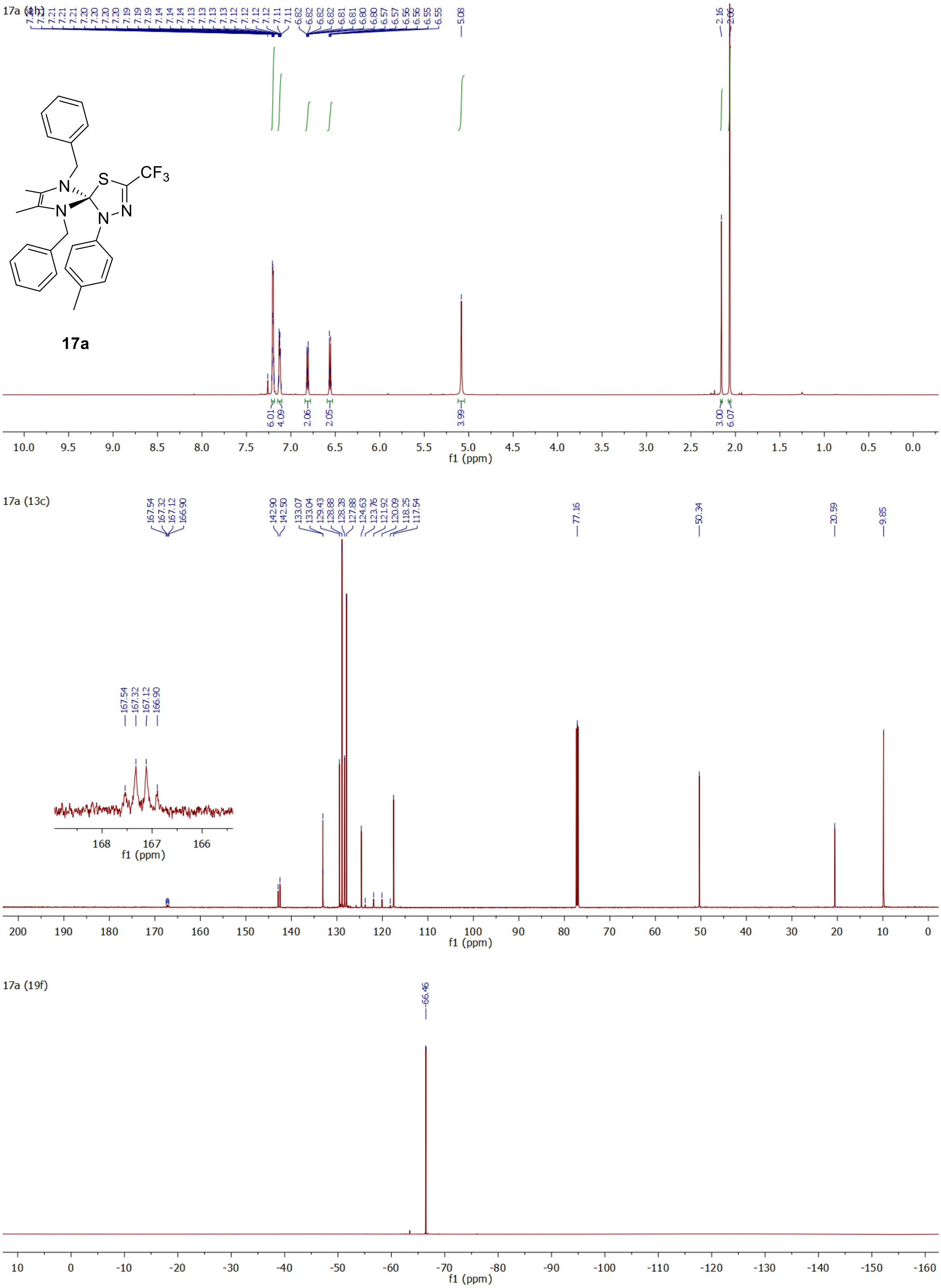

Figure S10. ^{1}H NMR (600 MHz, $CDCl_3$), ^{13}C NMR (151 MHz, $CDCl_3$) and ^{19}F NMR (565 MHz, $CDCl_3$) spectra for compound **17a**.

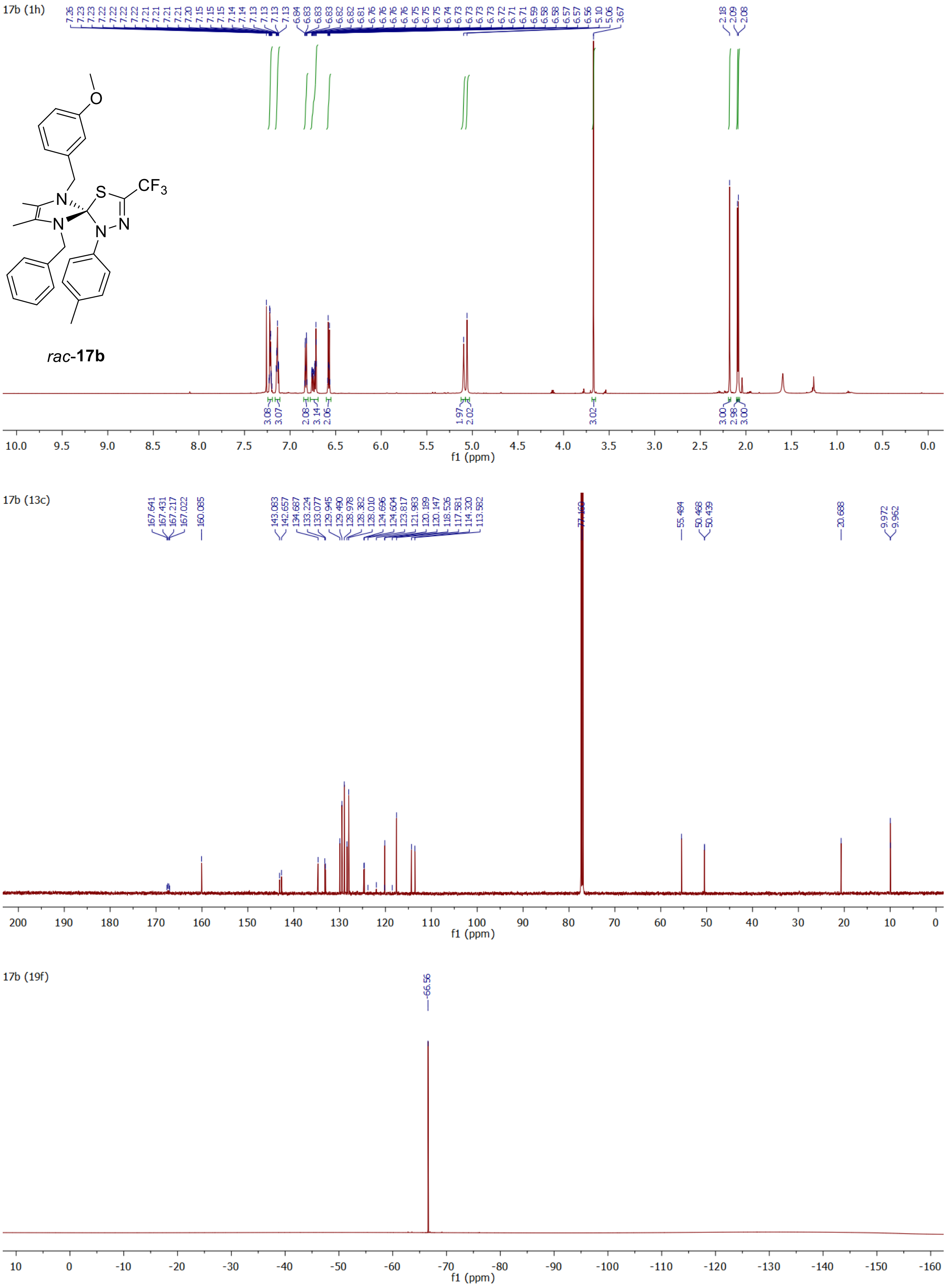

Figure S11. ^{1}H NMR (600 MHz, $CDCl_3$), ^{13}C NMR (151 MHz, $CDCl_3$) and ^{19}F NMR (565 MHz, $CDCl_3$) spectra for compound **17b**.

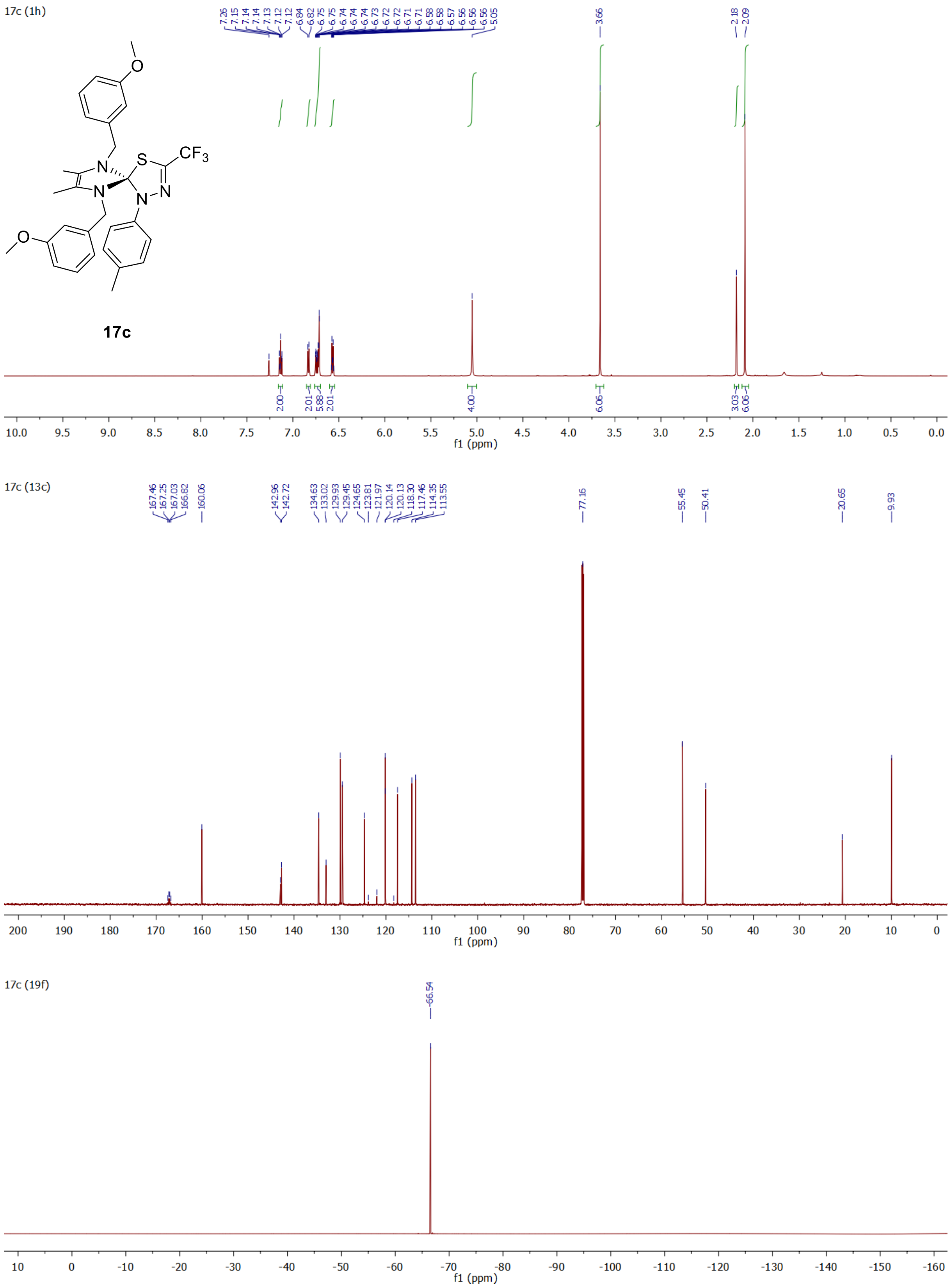

Figure S12. ^{1}H NMR (600 MHz, $CDCl_3$), ^{13}C NMR (151 MHz, $CDCl_3$) and ^{19}F NMR (565 MHz, $CDCl_3$) spectra for compound **17c**.

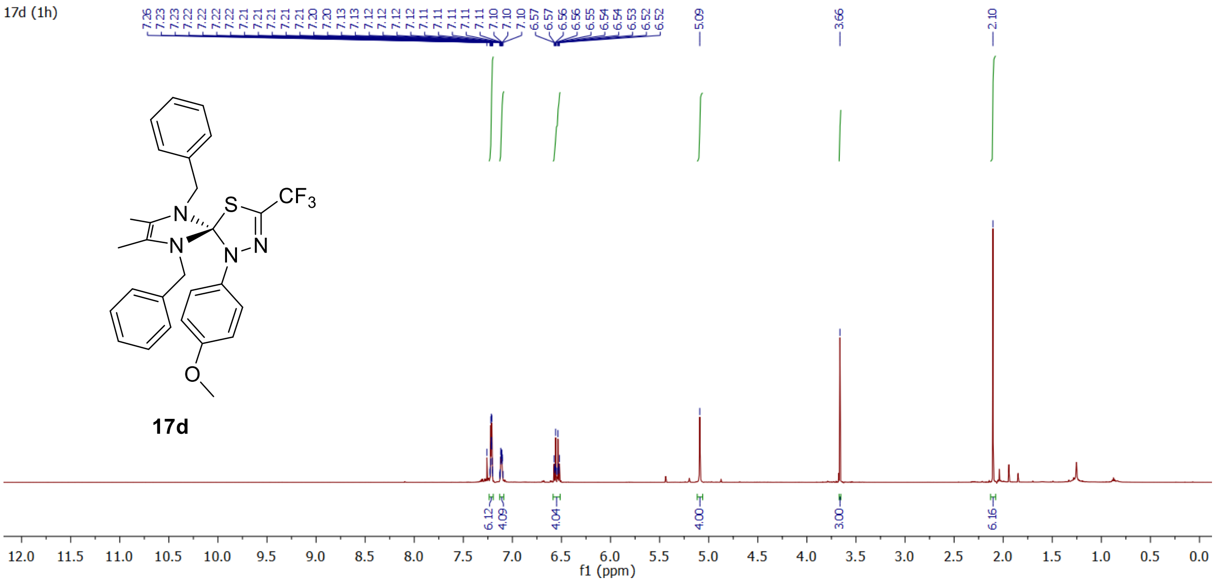

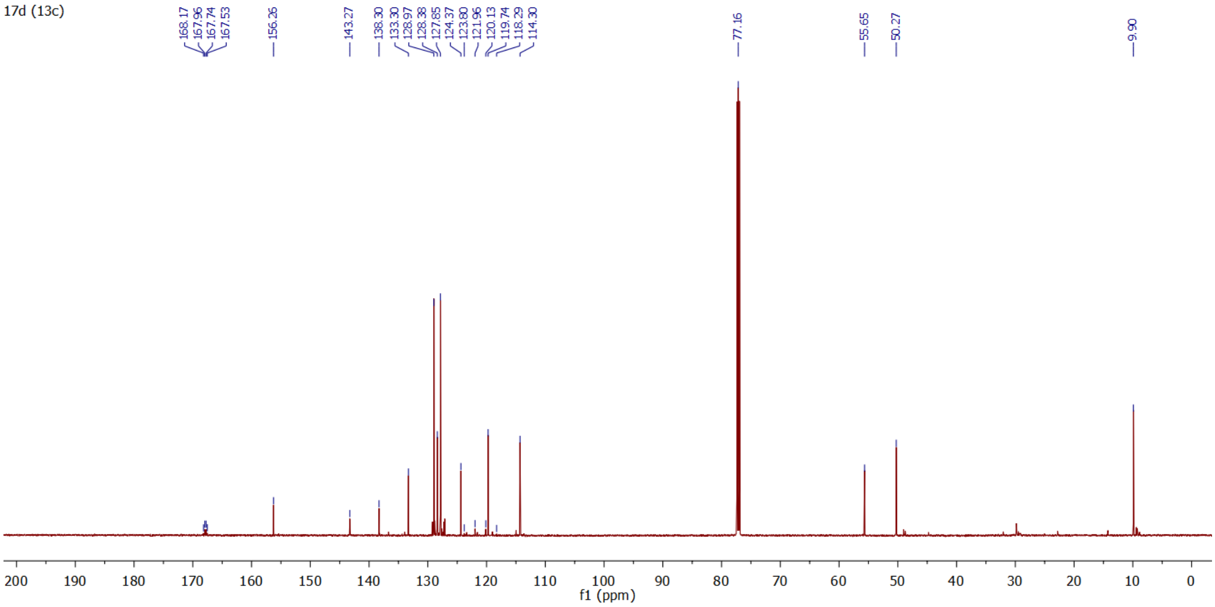

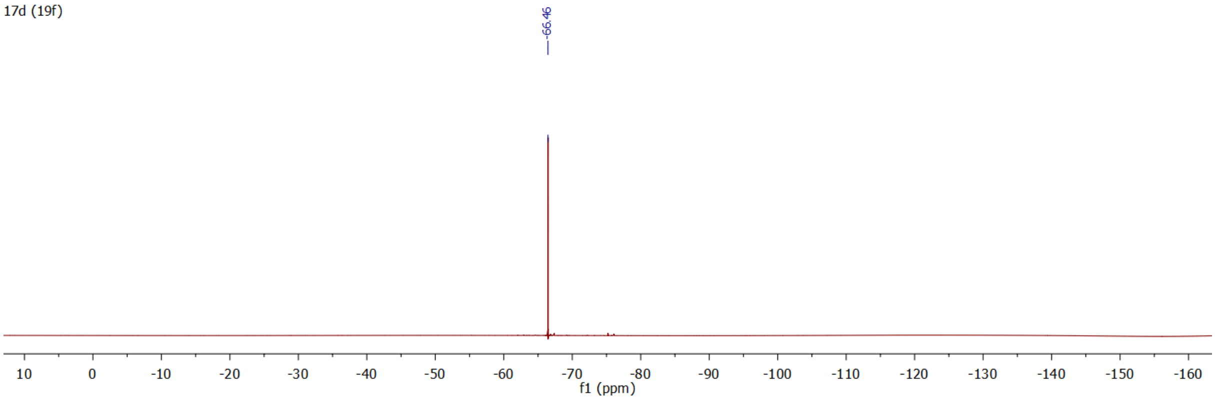

Figure S13. ^{1}H NMR (600 MHz, $CDCl_3$), ^{13}C NMR (151 MHz, $CDCl_3$) and ^{19}F NMR (565 MHz, $CDCl_3$) spectra for compound **17d**.

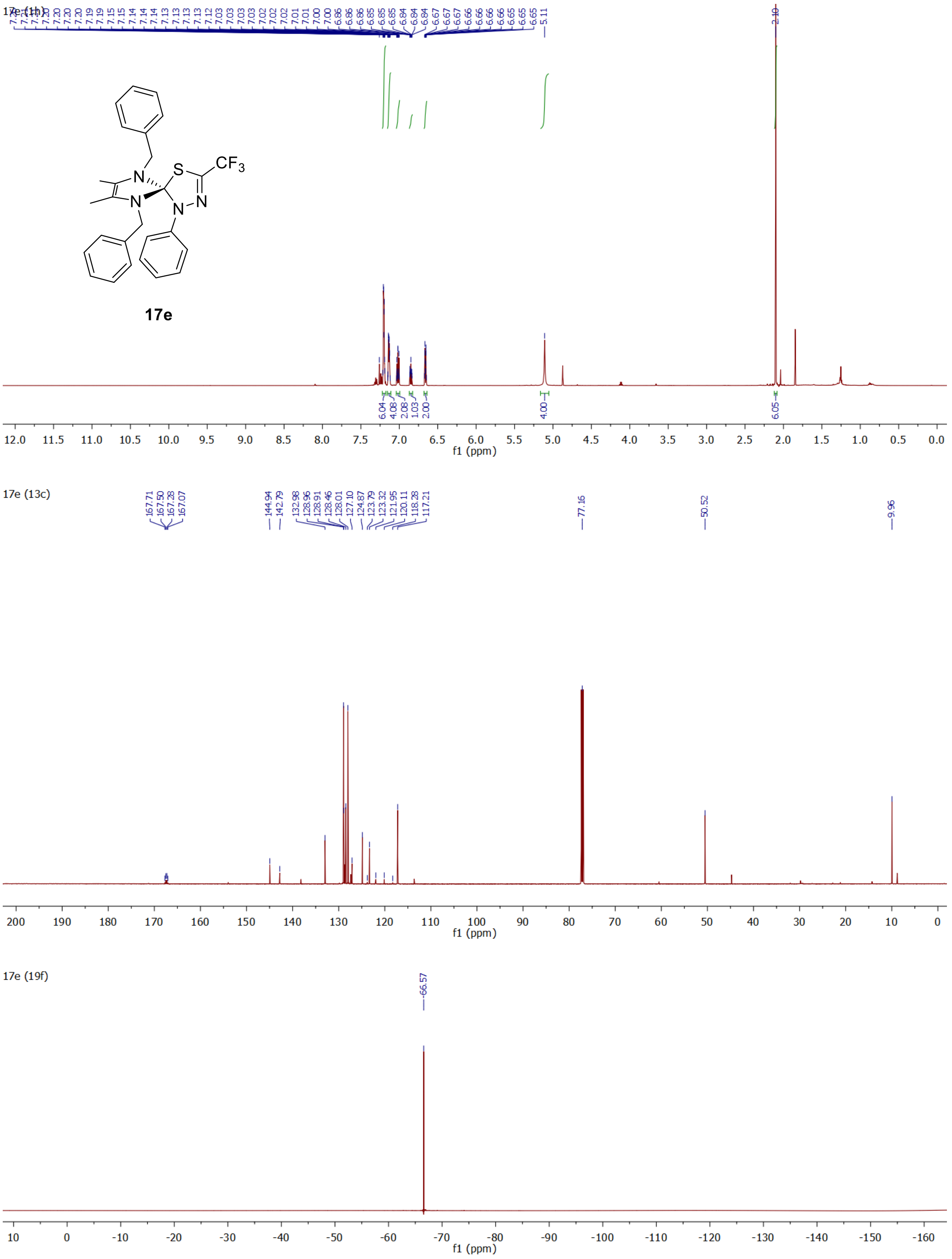

Figure S14. ^{1}H NMR (600 MHz, $CDCl_3$), ^{13}C NMR (151 MHz, $CDCl_3$) and ^{19}F NMR (565 MHz, $CDCl_3$) spectra for compound **17e**.

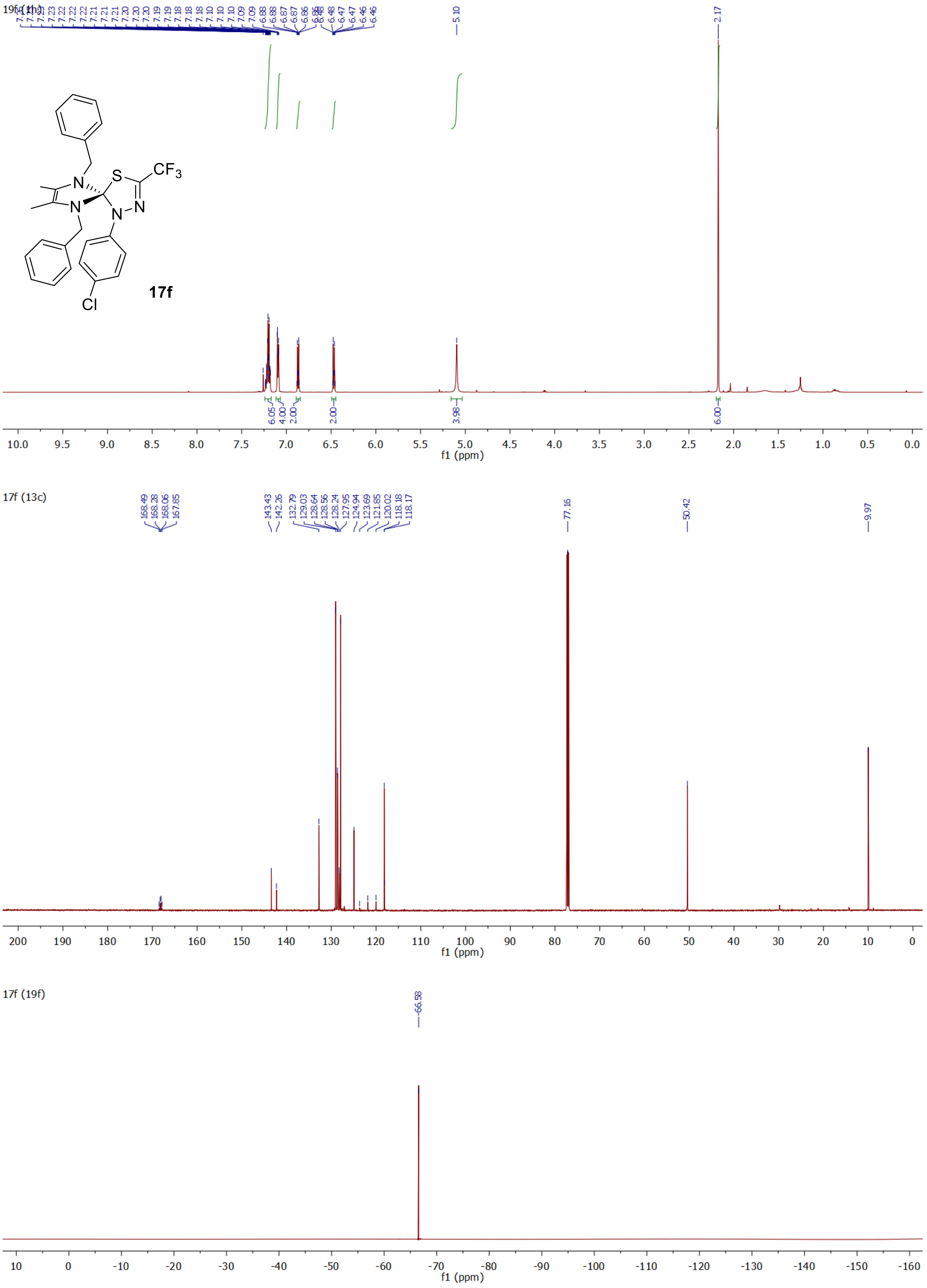

Figure S15. ^{1}H NMR (600 MHz, $CDCl_3$), ^{13}C NMR (151 MHz, $CDCl_3$) and ^{19}F NMR (565 MHz, $CDCl_3$) spectra for compound **17f**.

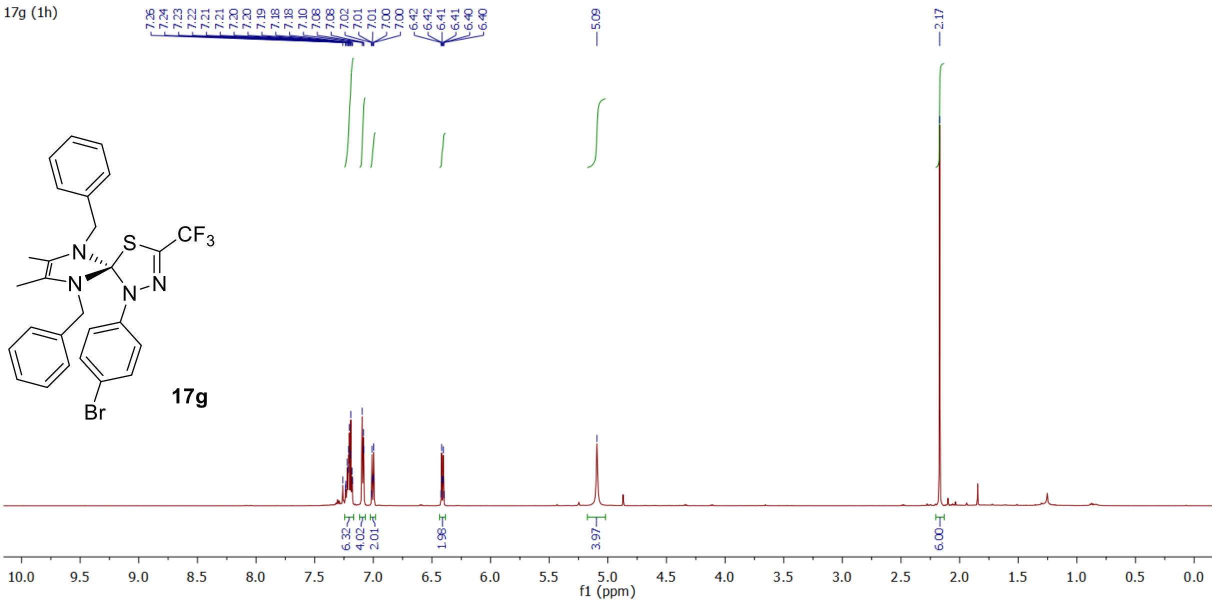

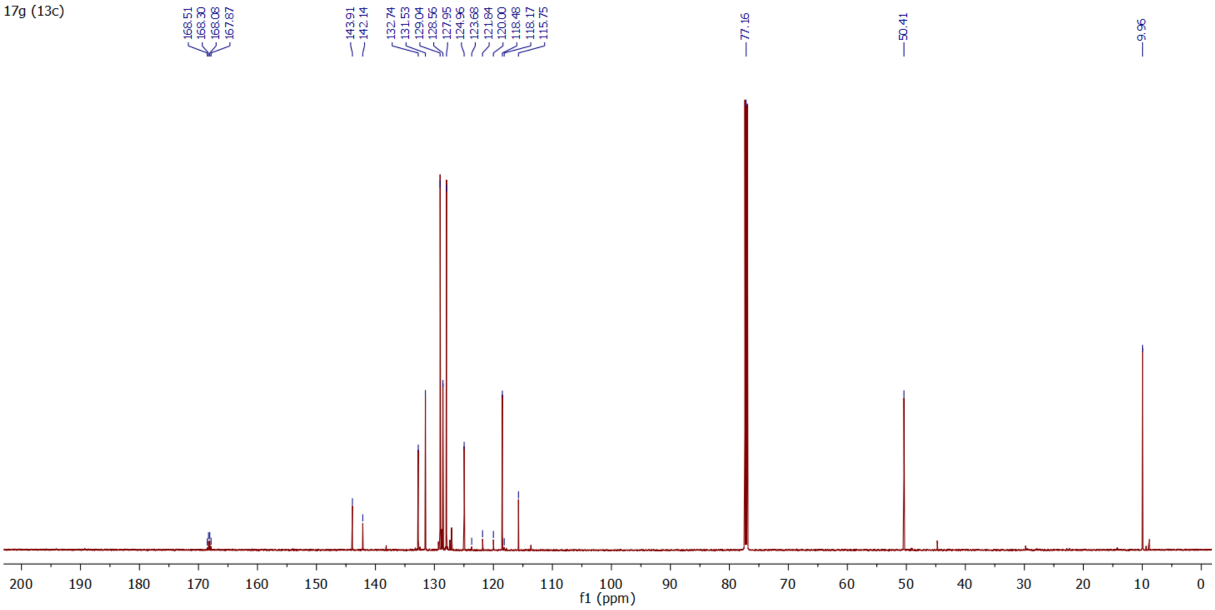

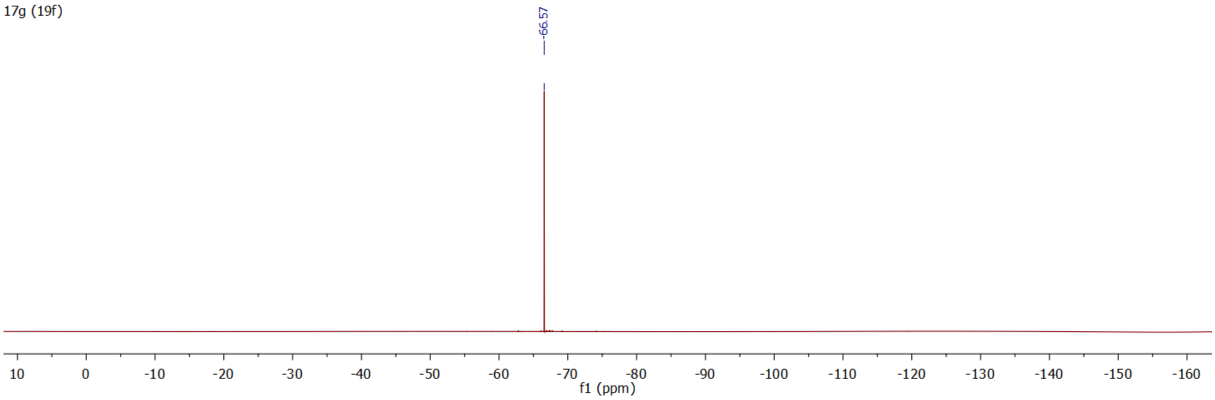

Figure S16. ^{1}H NMR (600 MHz, $CDCl_3$), ^{13}C NMR (151 MHz, $CDCl_3$) and ^{19}F NMR (565 MHz, $CDCl_3$) spectra for compound **17g**.

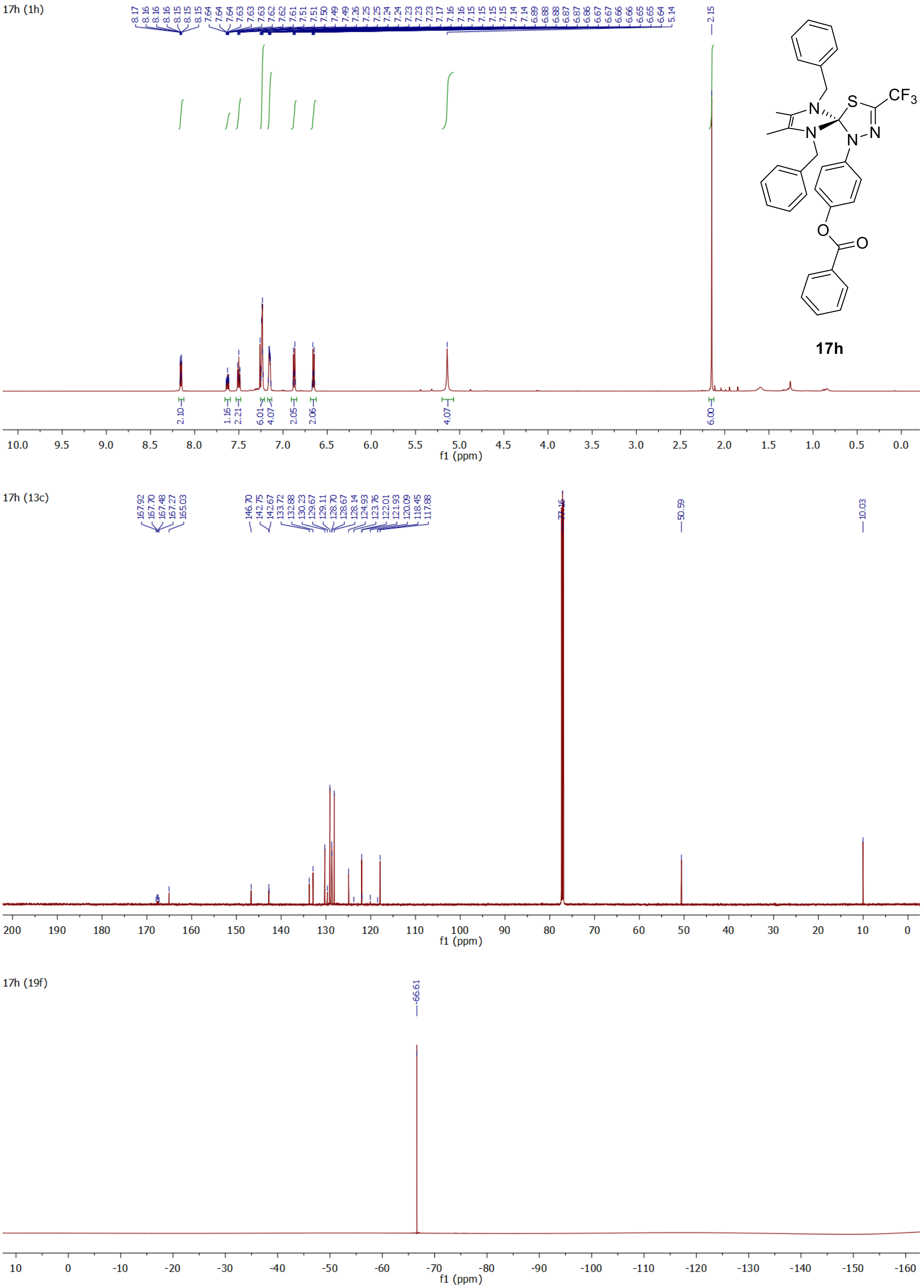

Figure S17. ^{1}H NMR (600 MHz, $CDCl_3$), ^{13}C NMR (151 MHz, $CDCl_3$) and ^{19}F NMR (565 MHz, $CDCl_3$) spectra for compound **17h**.

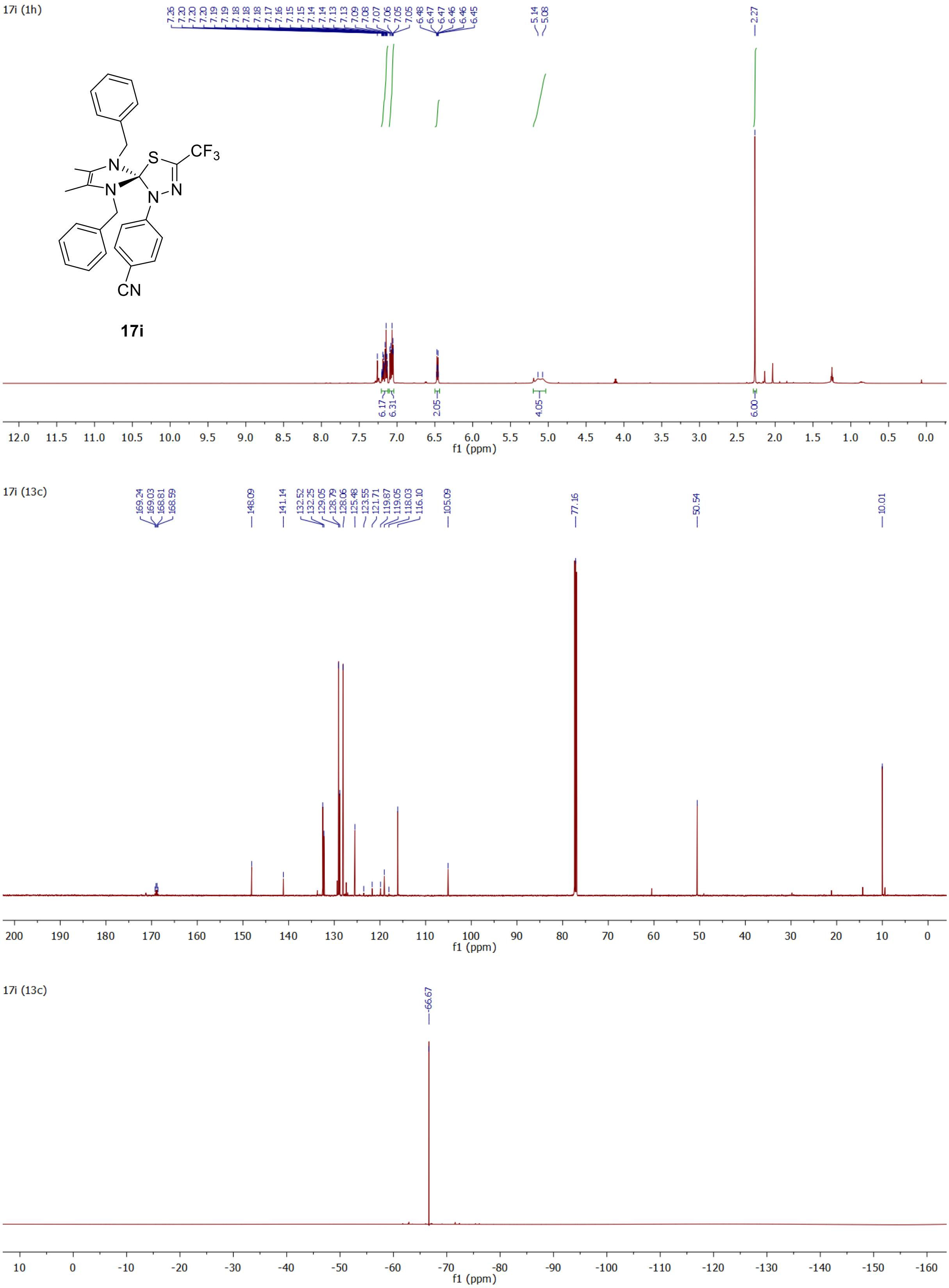

Figure S18. ^{1}H NMR (600 MHz, $CDCl_3$), ^{13}C NMR (151 MHz, $CDCl_3$) and ^{19}F NMR (565 MHz, $CDCl_3$) spectra for compound **17i**.

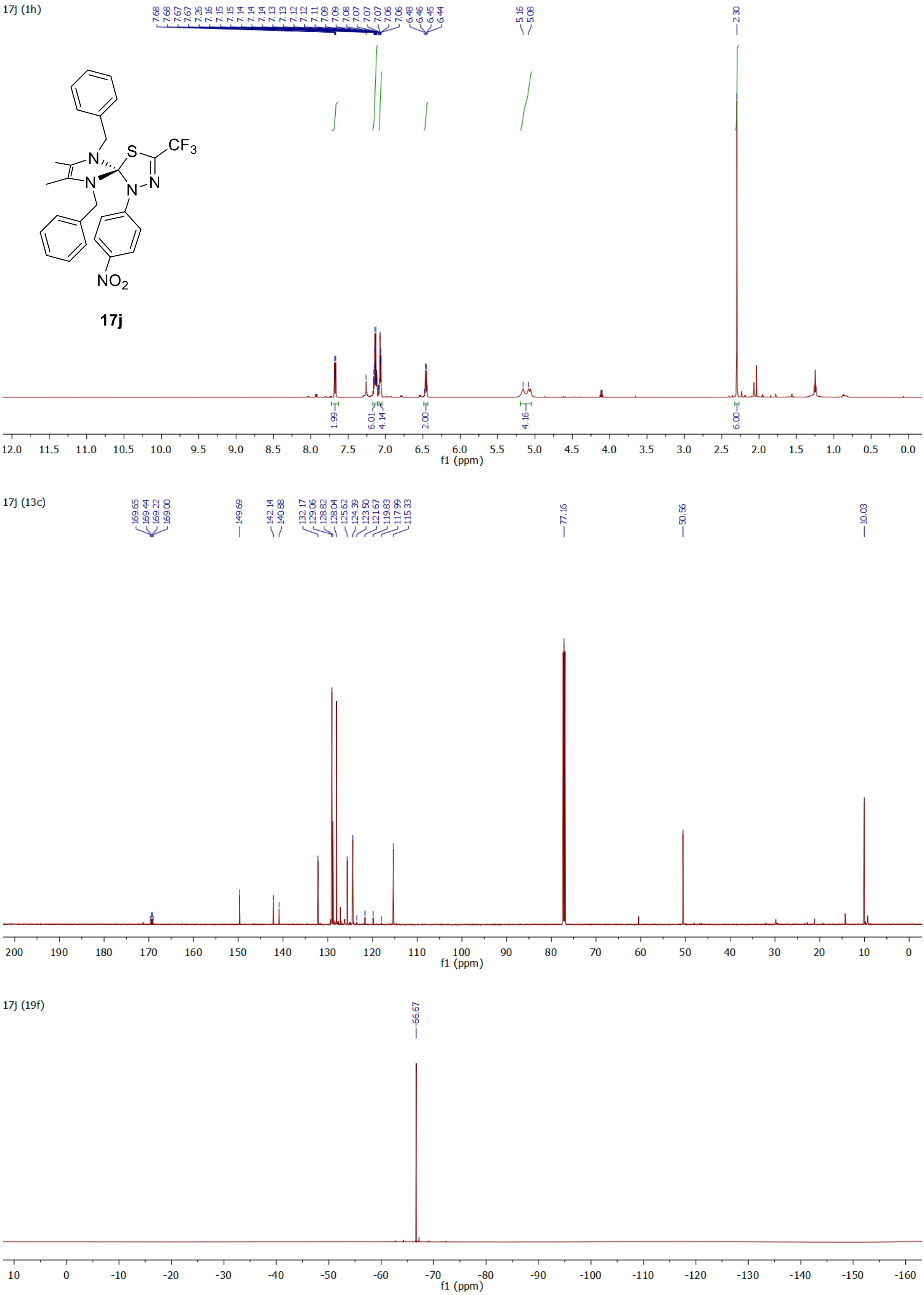

Figure S19. ^{1}H NMR (600 MHz, $CDCl_3$), ^{13}C NMR (151 MHz, $CDCl_3$) and ^{19}F NMR (565 MHz, $CDCl_3$) spectra for compound **17j**.

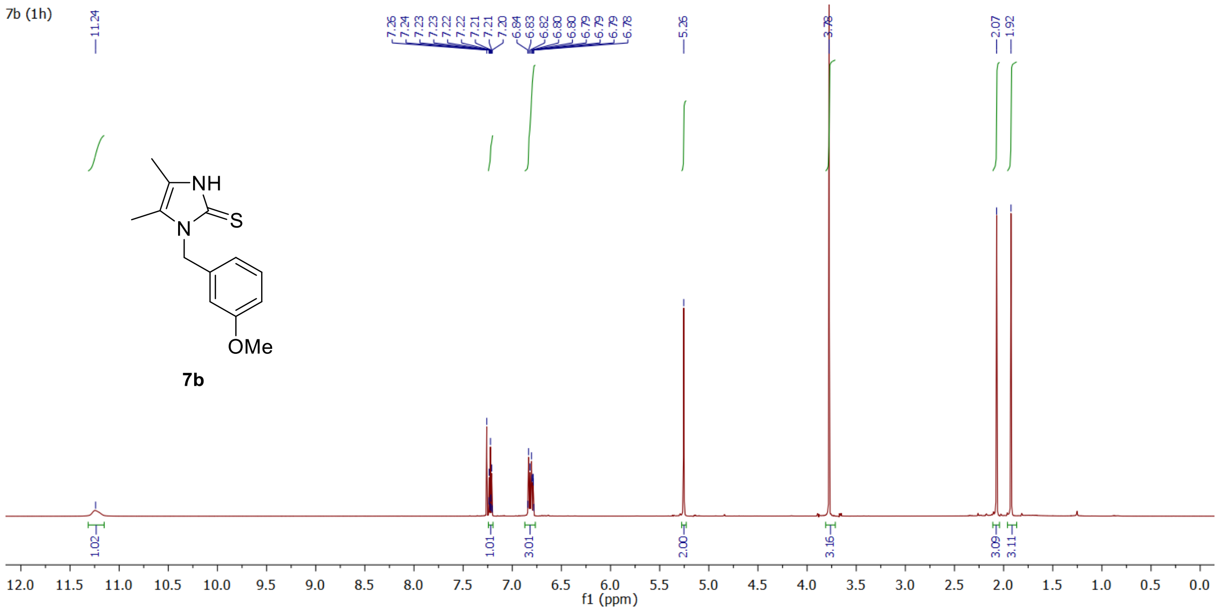

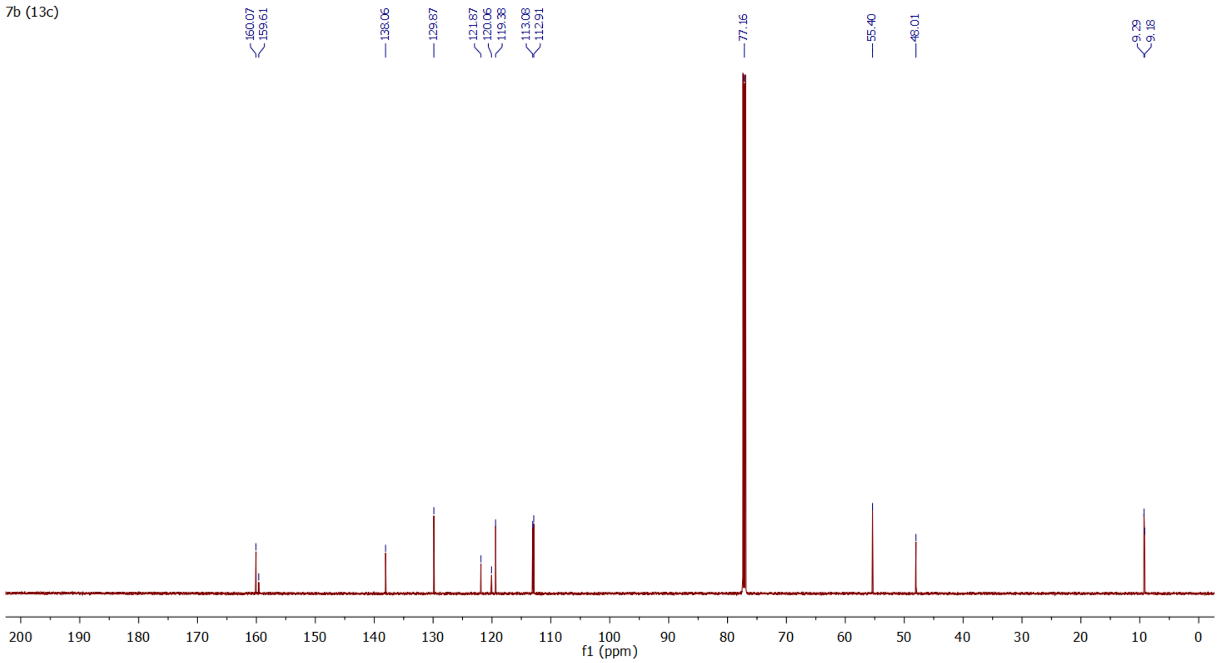

Figure S20. ^{1}H NMR (600 MHz, $CDCl_3$) and ^{13}C NMR (151 MHz, $CDCl_3$) spectra for compound **7b**.

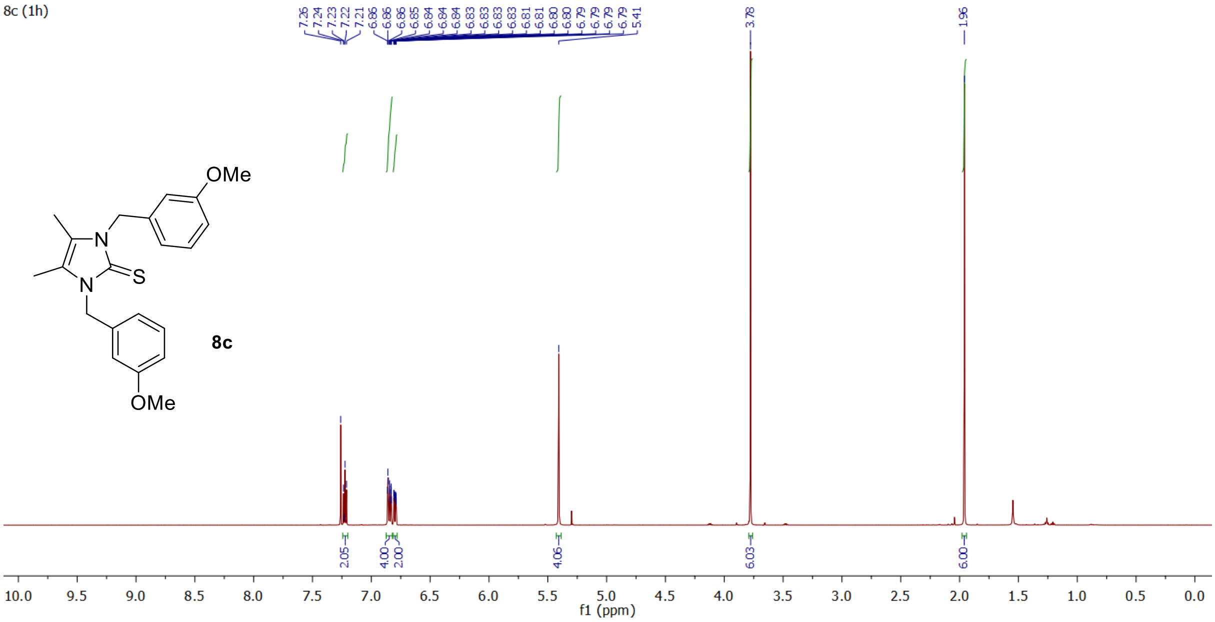

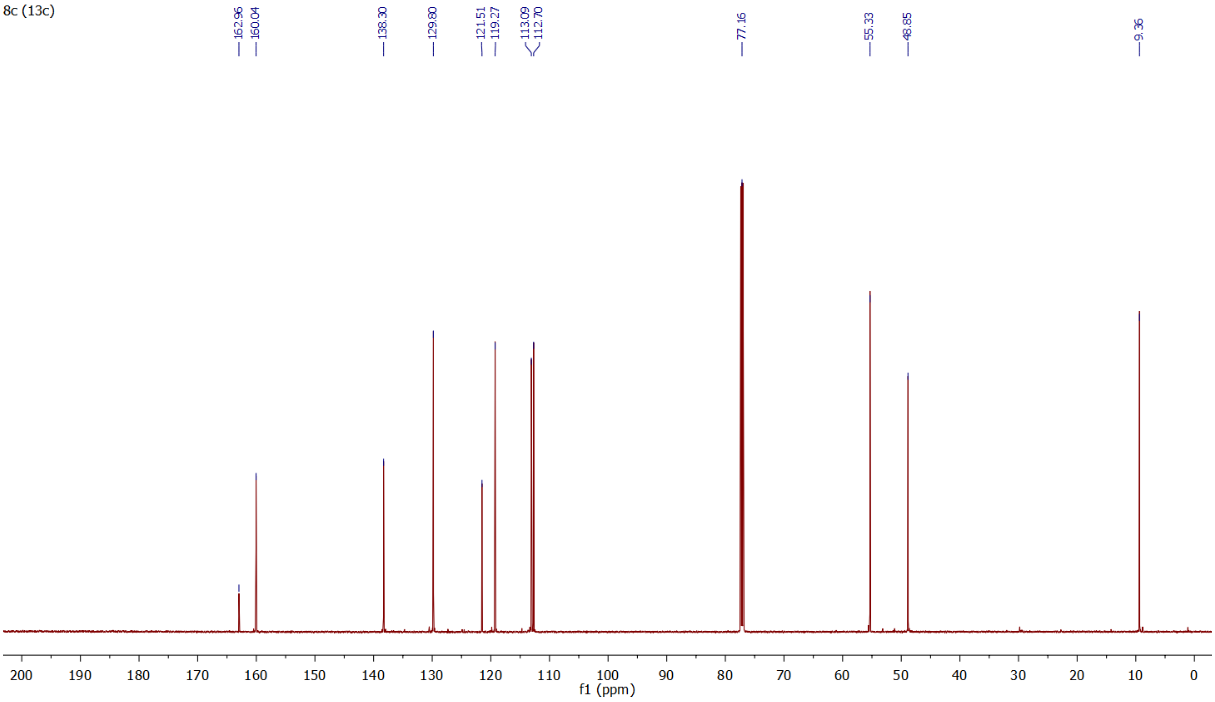

Figure S21. ^{1}H NMR (600 MHz, $CDCl_3$) and ^{13}C NMR (151 MHz, $CDCl_3$) spectra for compound **8c**.
